# Chromatin analysis of adult pluripotent stem cells reveals a unique stemness maintenance strategy

**DOI:** 10.1126/sciadv.adh4887

**Published:** 2023-10-06

**Authors:** Axel Poulet, Arcadia J. Kratkiewicz, Danyan Li, Josien C. van Wolfswinkel

**Affiliations:** ^1^ Department of Molecular Cellular and Developmental Biology, Yale University, New Haven, CT 06511, USA.; ^2^ Yale Stem Cell Center, Yale School of Medicine, New Haven, CT 06511, USA.; ^3^ Yale Center for RNA Science and Medicine, Yale School of Medicine, New Haven, CT 06511, USA.

## Abstract

Many highly regenerative organisms maintain adult pluripotent stem cells throughout their life, but how the long-term maintenance of pluripotency is accomplished is unclear. To decipher the regulatory logic of adult pluripotent stem cells, we analyzed the chromatin organization of stem cell genes in the planarian *Schmidtea mediterranea*. We identify a special chromatin state of stem cell genes, which is distinct from that of tissue-specific genes and resembles constitutive genes. Where tissue-specific promoters have detectable transcription factor binding sites, the promoters of stem cell–specific genes instead have sequence features that broadly decrease nucleosome binding affinity. This genic organization makes pluripotency-related gene expression the default state in these cells, which is maintained by the activity of chromatin remodelers ISWI and SNF2 in the stem cells.

## Teaser

Adult pluripotent cells regulate gene expression through chromatin remodelers, without relying on specific transcription factors.

## INTRODUCTION

Pluripotent stem cells are present in all metazoans. In many animals, and in particular in the most common animal model systems, such stem cells are restricted to the early stages of embryonic development. Most of our understanding of pluripotency thus relies on studies of embryonic (or induced) pluripotent cells from vertebrate models that naturally maintain these cells only for a brief period of time and in a closely protected environment. The image that has emerged from such studies is that of a genomically and epigenetically fragile cell state that strictly depends on a regulatory network of transcription factors (TFs) including OCT4 and SOX2 (*1*–*4*). In contrast to these common vertebrate models, many highly regenerative invertebrate animals retain pluripotent cells throughout adulthood. The ability to maintain stable pluripotent cells long-term would be highly beneficial for therapeutic applications, and it thus would be enlightening to learn from these animals that have long-lived pluripotent cells and investigate how they achieve this feat.

Planarians have emerged as a powerful model for stem cell biology. Planarian adult stem cells, known as neoblasts, have the ability to generate any cell type of the planarian body and are collectively and individually pluripotent (*5*). This capacity is used during regeneration to regrow any missing body parts, as well as during homeostasis to replace aged and damaged cells, giving these animals a possibly indefinite health span. The neoblasts thus are essential for planarian viability, and the lifelong maintenance of this pluripotent cell population is crucial to animal health.

Planarians encode a large set of genes that are highly enriched in expression in the stem cells and have been associated with the pluripotent state of these cells (*6*–*9*). In addition to the shared expression of these common neoblast genes, notable heterogeneity in gene expression was found to divide the neoblasts into multiple subclasses (*10*). Around 80% of neoblasts express sets of tissue-specific TFs in addition to the common neoblast genes (*10*–*13*), which is interpreted to mean that these cells are specified toward the respective tissue lineage. Single-cell sequencing studies and clustering approaches have provided increasing detail on such specified subclasses and their transcriptional markers (*13*–*15*). The undifferentiated pluripotent cells, however, appear to be largely characterized by the absence of tissue-specific gene expression. This indicates that the genes shared by all neoblast subpopulations are largely the same as the genes that are required for maintaining pluripotency. In particular, no TFs specific to the undifferentiated pluripotent state or to the neoblast population as a whole have yet been identified. Several factors that had initially been associated with the pluripotent neoblasts have since been found to be more sporadically expressed among pluripotent cells and to be enriched in the neuronal precursors instead (*11*). These studies therefore have not been able to resolve the question of how the pluripotent neoblast state is maintained.

Here, we took a complimentary approach to these earlier studies that analyzed neoblast-specific regulation. Instead of focusing on the TFs that are enriched in the stem cell population, we asked what regulatory elements are enriched in the stem cell genes. We analyzed the chromatin and genome organization of neoblasts and several isolated tissues to compare the chromatin and sequence context of neoblast-enriched genes to that of tissue-specific genes. For tissue-specific genes, we find clear evidence of regulation by a few master TFs that can activate large sets of tissue-specific genes. To our surprise, we find that neoblast genes do not show any signs of regulation by master TFs. Rather, we find that they contain signs of more global regulation by chromatin remodelers. Our analyses thus offer a new perspective on how the neoblast fate is maintained in these cells.

## RESULTS

### Tissue isolations identify stem cell genes, tissue-specific genes, and constitutive genes

**Fig. 1.**
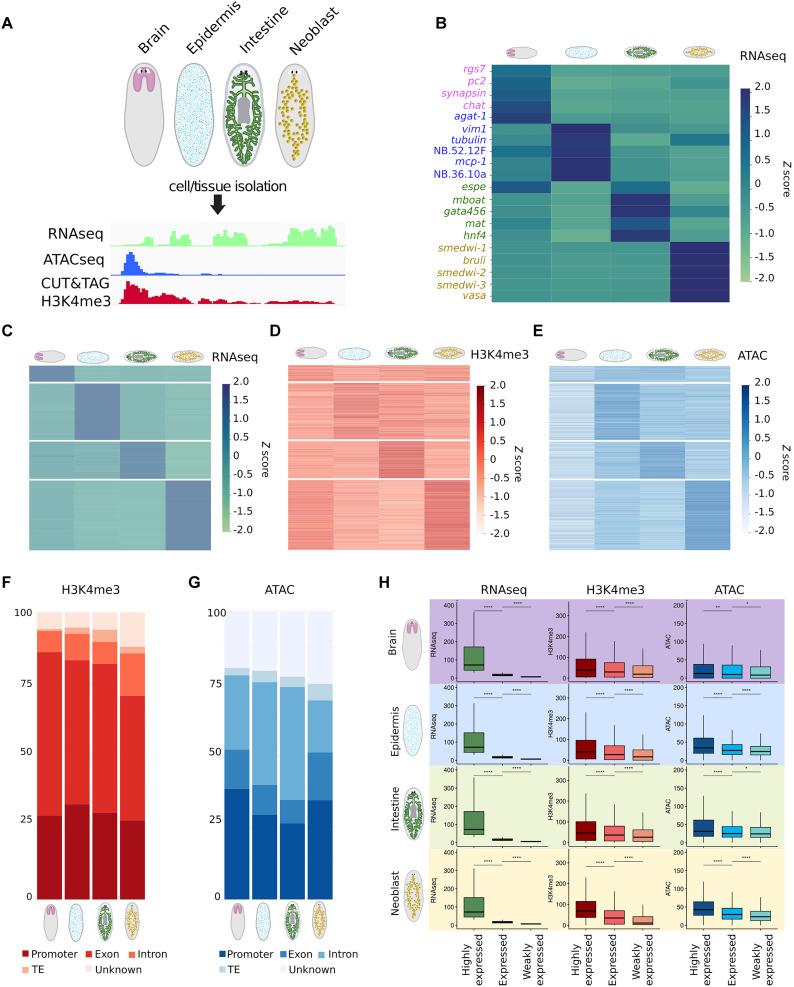
Tissue isolations identify tissue-specific and constitutive genes. (**A**) Schematic of the experimental design. (**B** and **C**) Heatmaps displaying normalized RNAseq data (B) of known tissue markers and (C) of all genes belonging to the specific clusters defined for each cell isolation. *Z* scores based on the transcripts per million (TPM), representing the relative expression levels of each gene across the different cell isolations normalized to the mean expression of the gene, are shown. (**D** and **E**) Heatmaps displaying (D) H3K4me3 CUT&Tag and (E) ATACseq. *Z* scores based on the reads per kilobase per million (RPKM) of the genes of the different tissue-specific clusters defined by RNAseq are shown. The gene order for (C) to (E) is identical. The RPKM for (D) and (E) was computed over a 2-kb region centered on the TSS. (**F**) Location of the H3K4me3 CUT&Tag peaks detected in each tissue. (**G**) Location of the ATACseq peaks detected in each tissue. Exons and introns are determined based on the mapping of poly-adenylated transcripts. The promoter is defined as the 2 kb upstream of the TSS. Transposable elements (TEs) were identified by RepeatMasker. (**H**) Analysis of the chromatin read coverage relative to the gene expression level. For each tissue, three gene categories were defined: highly expressed (>30 TPM), expressed (>10 TPM and <30 TPM), and weakly expressed (<10 TPM). Boxplots for the RNAseq (in TPM, in green), H3K4me3 CUT&Tag (in RPKM over the 2 kb centered on the TSS, in red), and ATACseq (in RPKM over the 2 kb centered on the TSS, in blue) are shown from left to right. Statistical significance was determined using a Wilcoxon test (*P* value with Bonferroni correction: *P ≤ 0.05, **P ≤ 0.01, ***P ≤ 0.001, ****P ≤ 0.0001).

To obtain robust transcriptomic and epigenomic data from different cell types, we isolated pools of neoblasts and pools of cells from brain, epidermis, and intestine through various sorting and dissection strategies (Fig. 1A; see Materials and Methods for details). We first generated RNA sequencing (RNAseq) libraries for each of the cell isolations. Analysis of differential gene expression confirmed that known markers of each tissue were highly enriched in the matching RNA sample, indicating the correct isolation of the intended specific cell populations (Fig. 1B). A further global analysis of the RNA content of the tissues identified clear clusters of neoblast- and tissue-specific genes (Fig. 1C; see Materials and Methods for details). Gene Ontology (GO) term analysis of each of the clusters of isolate-specific genes found enrichment of expected gene categories for each tissue and thereby confirmed the validity of the isolation methods (table S1).

In addition to the isolate-specific gene expression clusters, we also identified a set of genes that had similar expression levels in each of the isolates and we classified these as constitutive genes (fig. S1, A to C). We used publicly available single-cell RNAseq data to confirm the ubiquitous transcription of these genes including in cell types not isolated during our study (fig. S1D) (*16*). Furthermore, GO term analysis revealed an abundance of factors involved in essential cellular processes, including genes related to mitochondria, protein translation, proteostasis, and RNA processing (table S1). Together, these findings support their classification as constitutive genes, which we here define as genes that are expressed at similar levels in all planarian cells.

Next, we applied CUT&Tag (Cleavage Under Targets and Tagmentation) to determine the localization of the histone modification H3K4me3, which marks active promoters (Fig. 1D). CUT&Tag was recently developed as a more rapid and sensitive alternative to chromatin immunoprecipitation sequencing (ChIPseq) (*17*). To benchmark this method for detection of H3K4me3 in planarian cells, we compared our CUT&Tag data to publicly available ChIPseq data (*18*) and found strong correlation between the shapes of the peaks and the locations of the peaks across the planarian genome (fig. S1E). We expected that genes identified as having enriched expression in a specific cell isolation would also tend to show increased H3K4me3 signal in that same cell isolation compared to the other samples. It is important to note that this correlation is not expected to be perfect, as there are many more aspects involved in the activation of a gene than solely the H3K4 methylation state. We nevertheless inspected individual genes (fig. S2) and plotted the H3K4me3 signal for the gene clusters found in the isolate-specific RNAseq (Fig. 1D) and found that the expected trend is detected. Furthermore, a quantitative comparison of the RNA expression bins of genes and the strength of the H3K4me3 signal showed that there also is a quantitative correlation between these features. To further verify our data, we inspected where our identified H3K4me3 peaks were located on the genome. Previous reports had shown that in planarian genes, the H3K4me3 signal is elevated on both the active promoter and the early parts of the gene (*18*, *19*). In agreement with this, we found that most of the H3K4me3 peaks are located in promoters and exons (Fig. 1F and fig. S1F).

Finally, we identified the accessible chromatin regions in each of the cell isolations using assay for transposase accessible chromatin sequencing (ATACseq), which isolates open chromatin by its accessibility to the transposase Tn5 (*20*). Similar to what was explained above for the H3K4me3, we inspected the chromatin accessibility for the gene clusters found in the isolate-specific RNAseq data (Fig. 1, E and H, and fig. S2). This demonstrated that the expected correlation between chromatin accessibility and gene expression can be detected from our data. Further, we expected the accessible chromatin to locate primarily to genes and their promoters. We found little chromatin accessibility in regions that encode transposable elements (TEs) or in regions that were not closely associated with genes (Fig. 1G and fig. S1G). The limited number of peaks that were detected at distant loci could well relate to gene enhancer regions, but associating these with the correct gene is complex as linear proximity does not always correlate with regulatory relationships between a gene and an enhancer. Somewhat surprisingly, we also found many peaks in intronic regions of genes, primarily in the intestinal and epidermal samples. To investigate the basis for this finding, we asked which genes were marked by chromatin accessibility on their introns. We found that intron accessibility within a gene strongly correlated with increased chromatin accessibility at its promoter region (fig. S1, H and I). An intriguing possibility therefore is that these accessible intron regions can function as enhancer elements to the genes in which they are located and thereby enhance the effect of the promoter opening at these genes. To investigate the higher abundance of intron peaks in intestinal and epidermal samples compared to other cell isolations, we further inspected the intron content of the genes that are part of the isolation-specific gene sets (fig. S1, J to M). We found that in general, weakly expressed genes [1 to 10 transcripts per million (TPM)] had a higher intron content than highly expressed genes, but that, in addition to that trend, intestinal and epidermal genes tended to be larger and contain more and longer introns than neoblast- or brain-specific genes. The higher abundance of intron-localized ATAC peaks in intestine- and epidermis-enriched genes therefore may well be related to the higher amounts of intronic sequence in these gene sets.

Together, we find that the obtained datasets of RNA and chromatin features show the overall characteristics that would be expected based on prior knowledge of planarian tissues and of gene regulation in general. We therefore conclude that the obtained datasets are of sufficient enrichment and quality to use for analyzing the regulation of gene expression.

### Chromatin organization of constitutive genes is notably distinct from that of tissue-specific genes

**Fig. 2.**
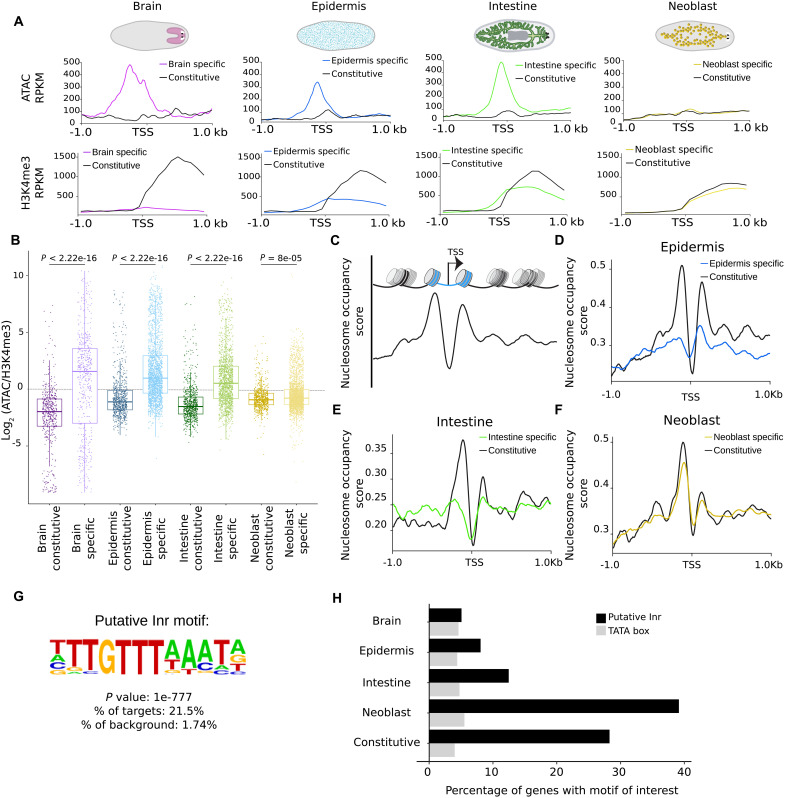
Chromatin organization of constitutive genes is notably distinct from that of tissue-specific genes. (**A**) Metaplots showing distribution of ATAC and H3K4me3 reads over the TSS (±1 kb) region of tissue-specific and constitutive genes in brain, epidermis, intestine, and neoblast (in RPKM). Gene counts used for this analysis are listed in table S5. (**B**) Boxplots comparing the distribution of log_2_(ATAC/H3K4me3) for tissue-specific and constitutive genes in brain, epidermis, intestine, and neoblast. Statistical significance was determined using a Wilcoxon test. *P* values are as indicated in the panel. (**C**) Schematic of the nucleosome occupancy. (**D** to **F**) Metaplots depicting nucleosome occupancy signal computed with nucleoATAC (*83*) centered on TSSs of the tissue-specific (colored lines) or constitutive (black lines) genes. (**G**) Putative Initiator (Inr) consensus sequence detected over the TSS (±40 nt). (**H**) Percentage of genes containing this putative Inr motif and TATA box over the TSS (±40 nt) in the different gene clusters (as determined by HOMER).

While H3K4me3 and ATAC levels globally correlated with RNA expression levels in each of the tissues (Fig. 1H and fig. S2), we noticed a pronounced distinction between tissue-specific genes and constitutive genes (Fig. 2A). In each of the isolated tissues, the ATACseq signal on tissue-specific genes showed a strong peak, which was located just 5′ of the transcriptional start site (TSS), at the site of the putative promoter region. In contrast, the constitutive genes showed only mildly elevated accessibility that overlapped with the TSS, and lacked the strong upstream peak of the tissue-specific genes. The tissue-specific genes showed a notable increase in H3K4me3 around the TSS, which peaked some distance into the genic region before returning to baseline level. The constitutive genes showed a similar distribution of H3K4me3 signal, although they reached a substantially higher intensity even when transcriptional output was comparable (Fig. 2A and fig. S3, A and B). The distribution of the ratios between H3K4me3 signal and ATAC signal for each individual gene confirmed this trend and showed that this reflects a widespread distinction between these two types of promoters (Fig. 2B).

Comparison of the TSS between tissue-specific genes and constitutive genes provided further evidence of distinct chromatin organization. The TSS of an active gene is typically depleted of nucleosomes to allow entry of RNA polymerase II. This nucleosome-free region is then flanked by two well-positioned nucleosomes (Fig. 2C) (*21*–*23*). We identified such nucleosome positioning at the constitutive genes in each of the tissues (Fig. 2, D and E). The tissue-specific genes, however, showed much weaker positioning of the −1 and +1 nucleosomes. Exploring the sequence composition around the TSS, we also found a difference in the prevalence of the core sequence motifs at the promoters. TATA boxes were detected in only 5% of planarian promoters, which is comparable to what is found in other metazoans (*24*), and this frequency was consistent across constitutive and tissue-specific promoters. We, however, identified a new motif that is enriched right at the TSS, suggesting that this is the planarian version of the Initiator motif (Fig. 2G and table S3). This motif was found in around 10% of tissue-specific promoters, but in almost 30% of constitutive promoters (Fig. 2H), reinforcing the notion that the organization of constitutive promoters is distinct from that of tissue-specific promoters.

This identification of distinct promoter types for constitutive and tissue-specific genes is in line with findings in several other models. In yeast, early studies on nucleosome positioning had identified promoters with large nucleosome-free regions and clearly positioned nucleosomes further upstream, which typically corresponded to low-plasticity (“housekeeping”) genes, whereas promoters with more evenly positioned nucleosomes corresponded to more dynamically regulated genes (*25*, *26*). Further, while some metazoan promoters have a single localized TSS, others were found to have multiple alternative TSSs spread over a larger region, which was associated with low-plasticity genes (*27*, *28*). A recent study of *Caenorhabditis elegans* promoter regions found that constitutive genes have well-positioned nucleosomes around their TSS, and that this coincides with a 10-nucleotide (nt) periodicity of AT motifs in their sequence (*29*). We here find that constitutive genes in planarians have the same well-positioned nucleosomes, but do not show a 10-nt periodicity, indicating that they use a different mechanism to achieve a similar outcome.

In summary, we find that the chromatin accessibility of tissue-specific and constitutive gene promoters is very differently organized, and that while both promoter types are marked by H3K4me3, only tissue-specific promoters have upstream peaks in accessibility that are reminiscent of TF binding sites.

### Chromatin organization of neoblast-specific genes resembles that of constitutive genes

We were interested to find how the stem cell–specific genes compared to these two types of promoter organization. Given that the stem cells are a specific cell type, we expected to find an arrangement similar to the tissue-specific genes. In contrast, when we analyzed the H3K4me3 profile and the ATAC profile of the neoblast-enriched genes, we found no increase in chromatin accessibility 5′ of the TSS. Instead, we found a mild increase in accessibility centered on the TSS, similar to what we had identified for constitutive genes (Fig. 2A). The ratio of H3K4me3 over ATAC signal in the neoblasts was also similar between neoblast-specific genes and constitutive genes (Fig. 2B), indicating that this constitutive-like chromatin arrangement is present at a major fraction of the neoblast-specific genes. The nucleosome positioning around the TSS similarly mirrored that of constitutive genes, showing firmly positioned −1 and +1 nucleosomes (Fig. 2F), which differs from what tissue-specific genes had shown. Motif analysis around the TSS revealed a high prevalence (40%) of the putative Initiator motif, similar to the situation in constitutive promoters (Fig. 2H). These findings show that the chromatin organization of the neoblast genes resembles that of constitutive genes rather than that of the dynamically regulated tissue-specific genes.

To ensure that our findings concerning the accessibility profiles across the promoter regions of tissue- and stem cell–specific genes are not caused by technical artifacts, we ran several control experiments (fig. S4). First, to exclude the effect of sample preparation, we applied the same processing steps and incubation times on isolated epidermal cells as are used for the isolation of stem cells. This did not alter the accessibility profiles of the epidermal-specific promoters, and an upstream peak in accessibility corresponding to a putative TF binding site was clearly detected (fig. S4, D and E). Second, to exclude cell cycle–related effects, we repeated the analysis with stem cells in G_1_-G_0_ phase of the cell cycle (fig. S4, F and G). This cell population contains early differentiating cells in addition to the stem cells, and thus, accessibility peaks related to the various differentiated tissues can now be detected in this dataset. However, this isolation strategy did not change the accessibility profile of the neoblast-specific genes, and still no accessible region upstream of the TSS was identified. Third, we retrieved publicly available planarian ATACseq datasets and analyzed them using our pipeline to determine the accessibility profiles of tissue- and neoblast-specific genes (fig. S5) (*30*–*32*). Although these datasets were mostly derived from mixed cell populations, in all datasets we recovered the similarity of neoblast genes and constitutive genes, whereas tissue-specific genes continue to follow a different profile, thus confirming our findings (fig. S5). The fact that our main findings are reproduced in various other datasets that were generated by different methods and different laboratories strongly suggests that they reflect true biological findings.

We were interested to find whether this distinct organization of stem cell–specific genes is unique to the adult pluripotent stem cells of *Schmidtea mediterranea* or whether this could be a more general property of adult pluripotent stem cells. While tissue-specific data are not available in other highly regenerative model systems, single-cell RNAseq and whole-animal ATACseq data are available from the evolutionarily distant acoel *Hofstenia miamia*, which also has adult pluripotent cells (*32*, *33*). Analysis of these data revealed that in this animal the promoter organization of stem cell genes is also different from that of tissue-specific genes, and instead is similar to that of constitutive genes (fig. S6). This indicates that the arrangement we found is not unique to planarians, and it suggests that this phenomenon may be widespread among adult pluripotent stem cells.

### Stem cell genes and constitutive genes lack enriched TF binding sites

**Fig. 3.**
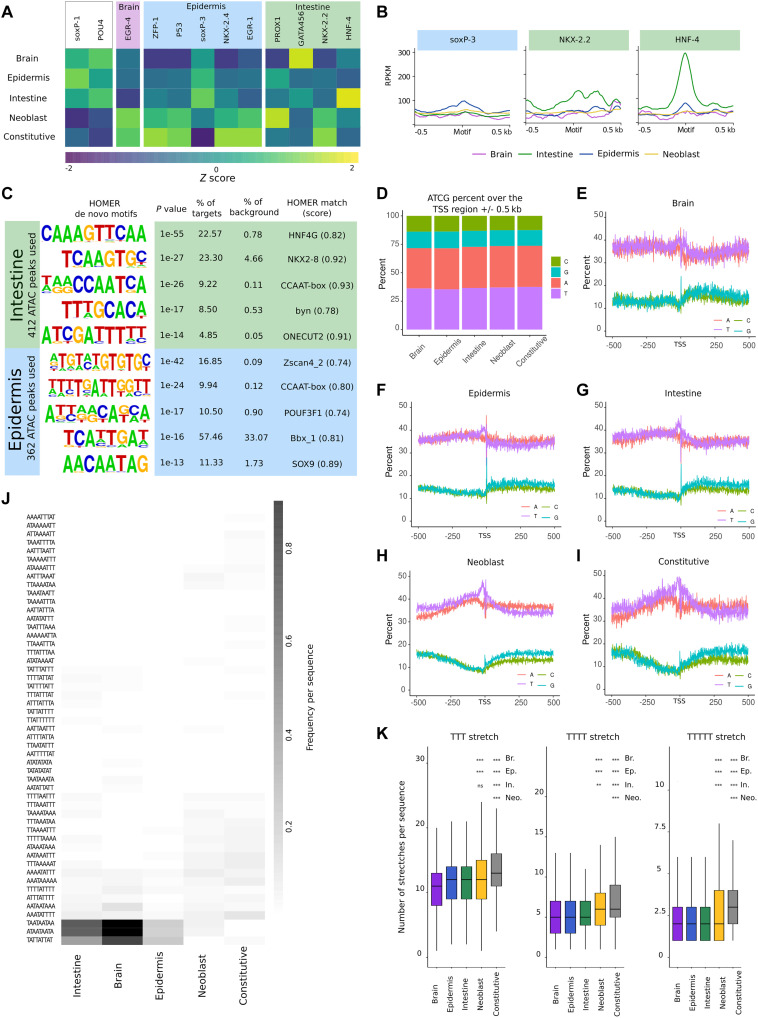
ATAC peaks reveal transcription factor motifs for tissue-specific genes, but not for neoblast genes. (**A**) *Z* score of the frequency of transcription factor (TF) motifs (see table S2) within the region 2 kb upstream the TSS as identified by FIMO (*92*). (**B**) Metaplots depicting chromatin accessibility over several motifs of interest from (A) (±500 b) in each of the tissues. (**C**) Enriched motifs in ATACseq peaks localized in the promoter regions of intestinal and epidermal genes. No significantly enriched motifs were found for neoblasts or constitutive genes. (**D**) Aggregated A, T, C, and G content of the region around the TSS (±500 b) in the different gene clusters. (**E** to **I**) Metaplots of the A, T, C, and G content in the region around the TSS (±500 b) in the genes specific to brain (E), epidermis (F), intestine (G), neoblast (H), or constitutive genes (I). (**J**) Analysis of nonamer enrichment in the 500 nt upstream the TSS. (**K**) Analysis of T stretch frequency in the 500 nt upstream the TSS. Statistical significance is shown for the neoblast and constitutive sets relative to the tissue-specific sets as determined by Wilcoxon test (*P* value with Bonferroni correction: ns: not significant, **P ≤ 0.01, ***P ≤ 0.001).

The promoter regions of the tissue-specific genes showed peaks in chromatin accessibility, which are expected to correspond to regions of TF binding. Previous studies identified several conserved tissue-specific TFs, and we confirmed the specific enrichment of several of these factors in the transcriptional data of our tissue isolations (Fig. 1B and fig. S7A). On the basis of their enriched expression levels, these TFs are expected to regulate gene expression in their respective tissues. We therefore explored whether binding motifs for the tissue-enriched TFs would be enriched in the promoters of the tissue-specific gene sets that we had identified. To evaluate this, we predicted binding motifs for several conserved, tissue-specific TFs (table S2) and scanned the general promoter regions (2 kb of sequence upstream of the TSS) of the tissue-specific genes for these motifs. Remarkably, we found no good correlation between the tissue of enrichment for a TF and the prevalence of the putative TF motifs in the general promoter regions of matching tissue-specific genes (Fig. 3A), reinforcing previous notions that the mere presence of a TF-binding motif upstream of a gene is not indicative of a regulatory interaction (*34*, *35*). We next used our tissue-specific ATACseq data to analyze the accessibility of the predicted TF-binding motifs in the promoter regions of the gene clusters (Fig. 3B and fig. S7B). This analysis showed increased accessibility of the putative matching motifs for several TFs specifically in the tissue in which they are expressed. For example, although motifs predicted to match the epidermal TF soxP-3 were most prominent in the promoters of intestinal genes, we detected an increased accessibility of these motifs only in the epidermal tissue. Similarly, we found increased accessibility of putative EGR-1 motifs in epidermis, EGR-4 motifs in brain, and putative GATA-4/5/6 and NKX-2.2 binding sites in the intestine. The specific accessibility of the intestinal TF HNF-4 was particularly clear. Furthermore, RNA interference (RNAi)–mediated knockdown of *hnf-4* confirmed the down-regulation of several predicted target genes as well as other intestinal genes (fig. S7C). Together, these findings indicate that our tissue-specific ATACseq data are able to identify regulatory motifs, and that motif accessibility is an essential feature in the consideration of regulatory interactions.

For some of the previously identified tissue-specific TFs, we did not detect tissue-specific enrichment of accessible binding motifs. As planarian TF-binding motifs have not been experimentally verified, we reasoned that this absence of detectable regulatory interactions could be due to the incorrect prediction of their binding motifs. Therefore, in an independent approach, we used HOMER for the de novo identification of any enriched motifs in the accessible areas of the isolation-specific genes, independent of TF annotation (Fig. 3C), and we subsequently annotated motifs based on the most similar motif as annotated in the HOMER database. We again recovered the putative HNF-4 binding motif and the NKX-2 binding motif in the intestinal samples, indicating that these binding sites are present and accessible in the promoters of a significant fraction of the intestinal genes, consistent with earlier reports proposing these genes as intestinal regulators (*10*, *36*). Other motifs that were enriched in intestinal genes include the CCAAT box, which is a common motif bound by NF-Y TFs, and motifs that are assigned to two additional TFs that have not previously been associated with the intestinal lineage (Fig. 3C).

Epidermal promoter regions also contained the CCAAT box. However, the primary motif enriched in epidermal promoters was predicted to correspond to a zinc finger protein. While it is not possible to predict which of the many planarian zinc finger proteins interacts with this motif, it is interesting to note that one of the primary epidermal TFs is the zinc finger protein ZFP-1 (*10*). The motif analysis also revealed a motif corresponding to SOX9 (or soxP-3), a TF that also was previously found to regulate the epidermal lineage in planarians (*10*, *37*).

The constitutive genes and stem cell genes did not have upstream ATAC peaks in their promoters, and the peaks at the TSS did not recover any enriched motifs other than the putative Initiator motif. As an additional strategy for these genes, we searched the accessible sequence within 2 kb upstream of the TSS, but neither of the strategies recovered significantly enriched motifs. We also inspected the nearby putative enhancer regions of stem cell genes for enriched TF motifs (fig. S8), and while this uncovered some mildly enriched motifs, they did not have a very well-defined signature, were not specific to the gene set, and were present only in a low fraction of regions. Together, this implies that there is no TF that drives expression of a significant proportion of neoblast-specific or constitutive genes.

### Promoters of stem cell genes and constitutive genes contain homopolymeric AT stretches

It is plausible that constitutive genes are not regulated by specific TFs, as they are expressed at similar levels at all times. Stem cell genes, however, were defined based on the fact that they are expressed at much higher levels in the stem cells than in any of the differentiated cell types, suggesting specific regulation. The absence of clear TF motifs was thus highly surprising. We therefore further inspected the promoter sequences to look for indications of regulation of the neoblast-specific genes. While no specific motifs were uncovered, we did detect an elevated AT content in these regions. The planarian genome has an overall AT content of around 70%, which is consistent with the overall AT level in the various promoter sequences (Fig. 3D). In the 500-nt upstream of tissue-specific TSSs, this AT content increases mildly (Fig. 3, E to G), but in the promoter regions of constitutive genes and stem cell genes, the AT content reaches levels of over 90% (Fig. 3, H and I). In addition, there is a notable bias for T over A in the region leading up to the TSS. No specific enriched patterns were found in these AT-rich regions, except for repeated instances of T-stretches of variable length and at variable position (Fig. 3, J and K, and fig. S9). Such homopolymeric dA:dT stretches were first reported in the promoter regions of several constitutive genes in yeast (*38*, *39*) and are generally enriched in eukaryotic genomes (*40*). While the mechanism of their effect on transcription has still not been fully resolved, they tend to have a positive effect on gene expression, reduce transcriptional noise, and increase accessibility (*25*, *41*).

Remarkably, we did identify enriched *k*-mers in the general AT-rich regions upstream of the TSS in the tissue-specific genes: Trinucleotide repeats consisting of A and T in various permutations were overrepresented in these promoters and were absent from constitutive promoters and stem cell promoters (Fig. 3J). This indicates that in contrast to the constitutive genes and stem cell genes, the promoters of planarian tissue-specific genes are rich in short tandem repeats. These motifs are not enriched in the accessible regions of these promoters and thus are unlikely to be related to TF binding. A recent *C. elegans* study also found certain dinucleotide repeats in the promoter regions of tissue-specific genes (*29*), and we found similar dinucleotide repeats in the putative enhancer regions of tissue-specific genes (fig. S8). The role of such nucleotide repeats is unclear, but in general, short tandem repeats have mutation rates that are orders of magnitude higher than other genomic sequences (*42*). They may thus contribute to the evolutionary dynamics of such regulated genes by altering the distance between TF binding sites and the TSS, thereby subtly altering the regulation of gene expression (*43*–*45*). We find that the most essential genes in the genome, the constitutive genes and the stem cell genes, were depleted of these mutagenic motifs.

Together, this shows that, also on the level of the genomic sequence, neoblast promoters resemble constitutive promoters and that these promoters have sequence features that may increase general accessibility without the action of TFs.

### Genomic A compartment is not enriched in promoters of stem cell or constitutive genes

In the absence of clear TF-mediated regulation, we wondered whether the spatial organization of the genome might direct the expression of stem cell genes. The planarian genome is rich in TEs, which make up over 50% of the genomic sequence (*46*) and are largely marked by histone modifications that are associated with heterochromatin (*30*). In the three-dimensional (3D) organization of the genome, such heterochromatic regions typically cluster together in certain domains of the nucleus, forming a heterochromatic compartment, or B compartment (*47*, *48*), whereas the active regions form nuclear domains of transcriptional activity, also referred to as A compartments (*48*, *49*). We asked whether the differential distribution of stem cell genes, constitutive genes, and tissue-specific genes across these compartments might explain the observed differences in their regulatory requirements.

**Fig. 4.**
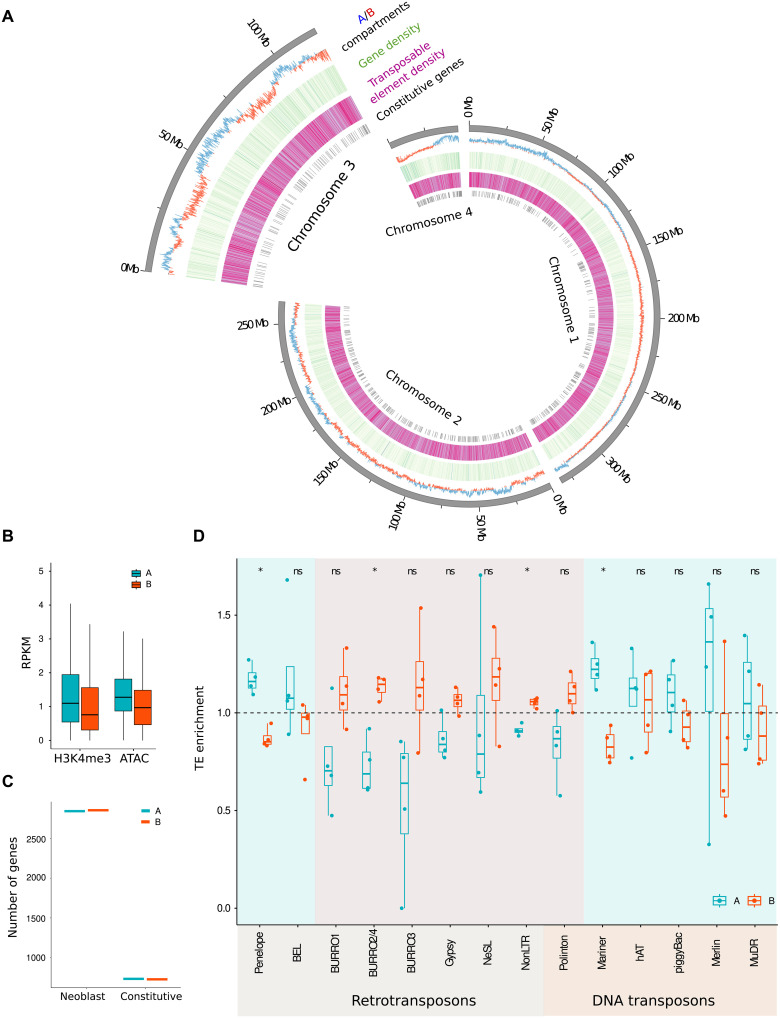
Genomic A compartment is not enriched in promoters of stem cell or constitutive genes. (**A**) Circos plot of the *S. mediterranea* genome organized per chromosome, showing A/B compartments (A in blue; B in red), gene density (light green for low, and dark green for high gene density), TE density (light purple for low, and dark purple for high TE density), and positions of the constitutive genes. (**B**) Comparison of the chromatin organization between the A and B compartment (A in blue; B in red). (**C**) Distribution of neoblast-specific genes and constitutive genes between the A and B compartments. (**D**) Enrichment in TE annotation per family over A/B compartment compared to the whole-genome percentage (set to 1.0). TE families are separated into retrotransposons and DNA transposons. Shading indicates the primary compartment for each family. Statistical significance is determined by Wilcoxon test (*P* value with Bonferroni correction: ns: not significant, *P ≤ 0.05).

We used publicly available Hi-C data to investigate the organization of such compartments in the planarian genome at 50-kb resolution. We found stable segregation of the genome into two compartments (Fig. 4A and fig. S10) (*50*). The A compartment showed higher accessibility than the B compartment, as reflected by higher levels of H3K4me3 and higher ATACseq read coverage, but we did not detect a difference in overall gene content, stem cell–specific gene content, constitutive gene content, or gene expression levels (Fig. 4, B and C, and fig. S10). Even when comparing these features between the two compartments within each chromosome (to avoid any potential chromosome-specific effects), we found no significant differences between the regions belonging to the two compartments (figs. S11 and S12). This finding was surprising, as studies in other model organisms have consistently found clear distinctions in genic content between the two main nuclear compartments. Many of these studies have been performed in mammalian systems, which tend to have larger genes than planarians, and thus may have larger linear sections of DNA segregating into the same compartment. It therefore cannot be excluded that planarian Hi-C analysis at higher resolution may reveal compartmentalization into domains that better correspond to gene expression. This is what was recently found in *Drosophila*, which has genes that are smaller than those of *Schmidtea*: Topologically associated domains (TADs) as resolved by high-resolution Hi-C data correlate much better with gene expression than the more global compartments (*51*). However, even *Drosophila* compartments called at low resolution did correlate with genic content (*48*). Therefore, our analysis suggests that the planarian compartments may function differently from the typical A and B compartments described in other model systems.

To better understand this lack of genic distinction between the compartments, we inspected the arrangement of genetic elements in the *S. mediterranea* genome (Fig. 4A). We find that the planarian genome is very dense in genetic elements, with relatively little noncoding space in between (median distance between elements is 1.08 kb). Further, genes and repetitive elements are intermingled throughout the entire genome, and we do not find extended regions that are depleted of genes or of repetitive elements. The neoblast genes and the constitutive genes are also not concentrated in specific regions of the genome, but rather are dispersed. Given this arrangement, it is likely that although some 50-kb bins are more accessible than others, most 50-kb regions will contain some genic as well as some repetitive elements. This indicates that no significant enrichment of genic elements in these compartments can be expected.

The only significant distinction in the sequence content of the compartments was found in the distribution of the TEs (Fig. 4D). The planarian genome contains over a dozen prominent families of TEs, and transposon copies of each family are distributed throughout the genome. We previously found that some families of TEs are actively repressed by Piwi-interacting RNA (piRNA)–mediated silencing and heterochromatin formation, whereas other families do not become active even in the absence of these silencing mechanisms (*30*). We found that the actively repressed transposons, including the retrotransposon families Burro and Gypsy as well as the replicative DNA transposon family of Polintons, were overrepresented in the B compartment on each of the chromosomes (Fig. 4D). Contrarily, the DNA transposons that do not require constant repression through piRNAs, such as Mariners and hATs, were enriched in A. This differential distribution of TEs supports the classification of the two compartments as functionally distinct and suggests that transposon content rather than genic content may drive the compartmentalization in *Schmidtea*.

Together, we detect interpretable compartmentalization of the planarian genome into A and B compartments. This large-scale compartmental organization, however, does not explain the difference in the regulation of the stem cell genes compared to the tissue-specific genes.

### Chromatin remodelers ISWI and SNF2 regulate stem cell gene expression

Classic in vitro studies proposed that poly(dA:dT) regions are rigid and free of nucleosomes (*52*–*56*), but a more recent study found that AT-rich DNA rather allows for the easy removal of nucleosomes by chromatin remodelers without affecting neighboring nucleosomes (*57*). We reasoned that in the absence of clear regulation by TFs or chromatin compartments, chromatin remodelers may play an important role in accomplishing the cell-specific activation of stem cell genes.

**Fig. 5.**
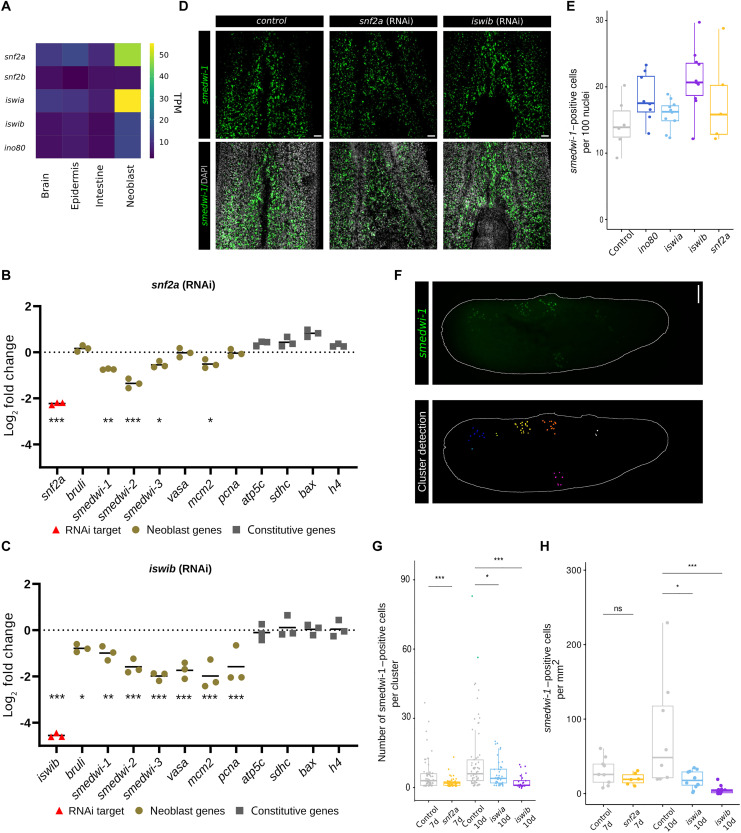
Chromatin remodelers ISWI and SNF2 regulate stem cell gene expression. (**A**) Heatmap of RNA expression levels of planarian chromatin remodeler genes. (**B** and **C**) Gene expression changes (qPCR) in neoblast genes and constitutive genes upon knockdown of chromatin remodelers *snf2a* (B) and *iswib* (C). Statistical significance is determined using a Student’s *t* test (*P ≤ 0.05, **P ≤ 0.01, ***P ≤ 0.005). (**D**) Fluorescent in situ hybridization of *smedwi-1* transcript, showing the number of neoblasts upon control RNAi treatment, or knockdown of *snf2a* or *iswib*. Scale bar, 50 μm. (**E**) Quantification of (D). (**F**) Illustration of the automated quantification of colony size. Different colonies as identified by the image analysis are marked in different colors. (**G** and **H**) Number of cells per expanding neoblast colony (G) and total neoblast density per animal (H) at 7 days (7d) after irradiation [*snf2a(RNAi)*] or 10 days (10d) after irradiation [*iswia(RNAi)* and *iswib(RNAi)*] compared to controls. Statistical significance is determined by Wilcoxon test (*P* value with Bonferroni correction: ns: not significant, *P ≤ 0.05, **P ≤ 0.01, ***P ≤ 0.001).

Of the four main types of chromatin remodelers, SWI/SNF, ISWI, and INO80 have been associated with altering accessibility at the TSS, whereas CHD-type remodelers typically modify accessibility in the gene body (*58*). Furthermore, previous studies proposed involvement of SWI/SNF-related complexes with planarian stem cell function, whereas a CHD remodeler was associated rather with cell differentiation (*59*, *60*). We identified one planarian homolog of INO80 and two homologs of each ISWI and SNF2 (fig. S13). In agreement with a previous characterization, we found that one of the SNF2 genes had very low expression (*59*), but expression of the remaining four genes was enriched in the stem cells (Fig. 5A). We used RNAi to knock down each of these genes and determined the effect on tissue-specific, stem cell–specific, and constitutive gene expression by quantitative polymerase chain reaction (qPCR) (Fig. 5, B and C, and fig. S14). *iswia(RNAi)* did not show any detectable effect on gene expression, and *ino80(RNAi)* mildly reduced RNA levels of stem cell and constitutive RNAs to the same extent. *iswib(RNAi)* and *snf2a(RNAi)* clearly resulted in a reduction of stem cell gene transcripts without significant effects on the constitutive genes (Fig. 5, B and C). To determine whether the reduction in stem cell gene expression could be due to a reduction in stem cell number, we quantified the percentage of total cells that were positive for the neoblast marker *smedwi-1* using quantitative analysis of whole-mount stainings (Fig. 5, D and E, and fig. S15A). We found no significant change in the neoblast abundance in any of our RNAi conditions, which is in agreement with a previous study that had found no reduction in stem cell number in the absence of SNF2a (*59*). Together, this indicates that the change in stem cell gene expression as detected upon reduction of ISWIb and SNF2a is due to a reduction in the level of stem cell transcripts per cell rather than to a change in cell composition of the animals.

One of the crucial tasks of planarian stem cells is to maintain the overall stem cell population and to repopulate the animal when stem cell numbers have been reduced. To test whether the detected reduction in stem cell gene expression is relevant for stem cell function, we tested the ability of the neoblasts to expand. We exposed the animals to a dose of radiation that significantly reduces stem cell number, and allowed the few remaining stem cells to divide and form colonies of neoblasts, which were detected by staining. To avoid any biases in the image analysis, we used an automated script (*61*) to determine the colony assignment of each neoblast and the number of cells per colony (Fig. 5, F and G, and fig. S15B). We found that knockdown of *iswib* and *snf2a* resulted in significantly smaller colonies than those found in control animals, indicating that in the absence of ISWIb or SNF2a neoblasts have a reduced activity and/or a reduced ability to maintain the neoblast fate (Fig. 5G and fig. S15B).

In summary, our data indicate that ISWIb and SNF2a maintain expression from stem cell genes in the absence of stem cell–specific TFs, and that their maintenance of stem cell gene expression is essential for stem cell function.

## DISCUSSION

**Fig. 6.**
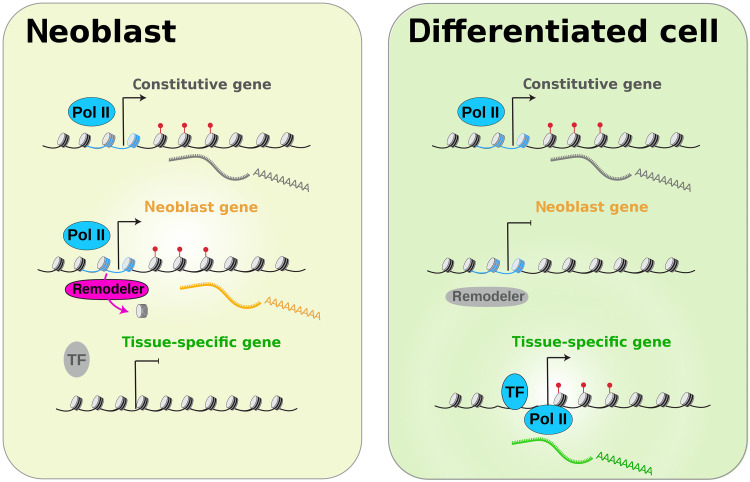
Model of neoblast gene regulation. Tissue-specific genes (**bottom**, green) are transcribed in differentiated cells (**right**) where they are marked by trimethylation of H3K4 (red). These genes are regulated by binding of tissue-specific TFs at a region of increased chromatin accessibility just upstream of the transcriptional start site (TSS). In the neoblasts (adult pluripotent stem cells, **left**), tissue-specific TFs are not present and the tissue-specific genes therefore remain silent. Neoblasts express a set of stem cell–specific genes (**middle**, yellow), and these are again marked by methylation of H3K4me3 (red). In contrast to the tissue-specific genes, activation of the neoblast genes does not depend on specific TFs, and no accessible regions upstream of the TSS are detected. Instead, the stem cell–specific promoters contain increased numbers of T-tracts, which decrease the affinity for nucleosomes. In the presence of chromatin remodelers ISWI and SNF2, this allows for the dynamic removal of weakly bound nucleosomes (blue) to provide access for polymerase II (Pol II) and activation of transcription. In the differentiated cells (right), the levels of these chromatin remodelers are much reduced and the neoblast genes remain silent. Constitutive genes (**top**, gray) are expressed in all cell types. These genes show promoter features similar to the neoblast genes but do not rely on the same chromatin remodelers for their expression.

Studies on embryonic pluripotent cells have shown that the pluripotent state is tightly regulated by a distinct TF network. Our findings here show that in contrast to that situation, planarian adult pluripotent stem cells are regulated in a manner that de-emphasizes the role of individual TFs (Fig. 6) and we find evidence that this organization is conserved in other adult pluripotent stem cells (fig. S6). The absence of clear TF binding peaks in the promoter regions of stem cell genes could mean that no TFs are bound, or alternatively, it could mean that many TFs can bind redundantly in various places and that their effects on the accessibility even out and no individual motifs are enriched. In either case, this means that the importance of individual TFs for the expression of stem cell genes is likely very limited.

At first glance, our findings put the adult pluripotent stem cells at odds with studies on mammalian embryonic pluripotent cells. Mammalian embryonic stem cells (ESCs) are tightly regulated by a core group of TFs, and the central role of the master regulator OCT4(*pou5f1*) in their maintenance is well established. The *pou5* branch of TFs that includes OCT4 arose from a gene duplication event that occurred within vertebrates (*62*, *63*), and it is therefore plausible that its pluripotency-related function is absent in invertebrate systems. One of the major functions of OCT4, however, is to recruit chromatin remodelers to stem cell genes to promote their expression (*64*, *65*). Remarkably, our data show that—even in the absence of OCT4—planarians recruit chromatin remodeling activity to promote pluripotency-related gene expression. Our findings thus emphasize the importance of chromatin remodelers in the regulation of pluripotent cells.

The finding that chromatin remodeling rather than the binding of a specific set of TFs activates the expression of the core stem cell genes is highly surprising given our understanding of gene regulation in general, but it does fit with other observations on stem cell RNA expression and fate specification in planarians. Single-cell studies on planarian neoblasts have identified sets of lineage-specific TFs in specified subsets of the neoblasts, but no specific TFs have been identified for the unspecified pluripotent neoblast cells, or for the neoblast population as a whole (*10*, *13*–*15*, *66*). On the basis of these single-cell analyses, it appears that the pluripotent state of the neoblast is mostly characterized by the lack of expression of tissue-specific TFs. Our finding that the core stem cell genes do not have clear TF binding sites supports the notion that there may not be any specific TFs to be found for the pluripotent state. The implication of our findings is that the expression of the core stem cell genes can be considered the default state of the neoblasts, and as long as chromatin remodelers are available to move the unstably bound nucleosomes out of the stem cell promoter regions, this expression state will be maintained (Fig. 6). A recent study described that planarian stem cells start to express tissue-specific TFs during S phase but revert to an unspecified state after mitosis when the tissue-specific transcripts have all been segregated into the differentiating daughter cell (*11*). Our data support the implication that the absence of tissue-specific TFs may be sufficient to default back to the pluripotent neoblast state.

Gene activation through chromatin remodelers seems rather aspecific, and our data cannot fully explain how stem cell–specific genes are switched off upon cell differentiation. We found that transcripts for several chromatin remodelers are more abundantly expressed in the stem cells than in differentiated cells. It is possible that the chromatin remodelers are actively retained at higher levels in the stem cell daughter cells or that they are regulated by other means. It is also possible that stem cell genes are actively silenced in differentiated cells by specific mechanisms, such as histone modifications, histone variants, or changes in 3D organization. Future exploration of these and other regulatory features will give insights into how planarian neoblasts exit the stem cell state.

## MATERIALS AND METHODS

### Experimental design

The primary objective of this study was to identify factors or mechanisms regulating the expression of stem cell genes in planarian neoblasts. As TF binding to promoter regions is a well-known mechanism of gene expression regulation, we generated several sequencing datasets to identify a set of genes with stem cell–specific expression and determine the chromatin organization of the promoter regions of these genes, with the goal of identifying TF binding sites. For comparison, we generated similar data for several differentiated tissues. We tested methods for the identification of TF binding sites from our datasets using the tissue-specific data and found that chromatin accessibility is an essential consideration in these analyses. We further implemented de novo motif enrichment analysis of tissue-specific accessible promoter regions to allow for the identification of binding sites for yet-uncharacterized TFs. When none of these strategies revealed TF binding sites for stem cell genes, we analyzed other sequence characteristics of the stem cell–specific promoters, as well as the role of 3D organization and of chromatin remodelers in stem cell gene expression.

### Experimental model and husbandry

*S. mediterranea* asexual clonal strain ClW4 was maintained as previously described (*67*). Briefly, animals were cultured in 1× Montjuic salts at 20°C, fed homogenized beef liver paste every 1 to 2 weeks, and expanded through continuous cycles of amputation or fissioning and regeneration. Animals were starved 1 to 2 weeks before experiments.

RNAi was used to accomplish knockdown of specific transcripts in planarian cells. For RNAi experiments, regions of planarian genes 0.5 to 2 kb in length were amplified from complementary DNA (cDNA) using sequence-specific primers (table S4) with adaptor sequences. The PCR product was cloned into the pGEM-T vector (Promega) and verified by Sanger sequencing. Both RNA strands were synthesized in vitro from PCR-generated forward and reverse templates with flanking T7 promoters (TAATACGACTCACTATAGG) and annealed by incubation at 37°C for 30 min. The transcribed single-stranded RNA (ssRNA) as well as the final double-stranded RNA (dsRNA) product were verified by gel electrophoresis. Animals were fed twice a week on liver containing in vitro synthesized dsRNA for 2 weeks, followed by starvation for 5 days before harvesting. In each experiment, the effectiveness of the gene knockdown was verified by qPCR.

Sublethal irradiation was used to strongly reduce the number of neoblasts per animal and study the expansion of the remaining neoblasts to obtain a more sensitive readout of their health. For sublethal irradiation experiments, animals were fed for three RNAi feeds over a course of 10 days and exposed to 1250 rads of irradiation on day 14. This reduces the number of neoblasts to only a few cells per animal. Surviving neoblasts were allowed to expand into colonies for 7 or 10 days before fixation and analysis.

### Genome and genome annotation

The chromosome-based assembly of the *S. mediterranea* genome (*50*) was annotated with merged transcriptome annotations (*10*, *68*, *69*). Transposons were annotated using RepeatMasker (http://www.repeatmasker.org).

### Fixation and whole-mount staining

Whole-mount staining of RNAi-treated animals was applied to detect neoblasts, to quantify the effect of chromatin remodelers on neoblast function. Fixations and whole-mount in situ hybridizations (ISHs) were performed as previously described (*70*), with alterations described in (*71*). Briefly, formaldehyde-fixed animals were bleached using formamide bleach solution and treated with proteinase K (2 μg/ml) in PBSTx (PBS with 0.1% TX-100). Probes were synthesized by in vitro transcription as digoxigenin (DIG) or fluorescein-labeled RNAs from constructs similar to the dsRNA constructs described above (see table S4 for primer sequences) and dissolved in formamide. Samples were hybridized with the probes overnight at 56°C. Subsequently, samples were washed sequentially in pre-hyb solution, 1:1 pre-hyb:2× SSC, 2× SSC, and 0.2× SSC at 56°C. Probes were detected with anti-DIG–POD (Roche) or anti-Fl–POD (Roche). After tyramide development (*71*), peroxidase was inactivated by incubation in 1% sodium azide.

### Planarian tissue and neoblast isolations

To compare the gene expression and chromatin organization between neoblasts and various differentiated tissue types, we isolated several cell populations.

Neoblasts in G_2_-M phase (X1) or G_0_-G_1_ phase (X2) and differentiated cells (Xins) were isolated by fluorescence-activated cell sorting (FACS) based on DNA content (Hoechst fluorescence) using gating as originally described by Hayashi *et al.* (*72*). Cells were prepared for FACS following the procedures described previously (*10*). Briefly, animals were dissociated in Calcium Magnesium Free buffer (CMF) supplemented with 1% bovine serum albumin and collagenase (1 mg/ml). Cells were passed through a 40-μm strainer and stained with Hoechst (0.4 mg/ml) and calcein (1 μg/ml) for 40 min at 20°C in the dark. No additional treatment was applied for RNAseq or for chromatin analysis.

Planarian brains used for RNAseq were isolated by fixation in 0.33 N HCl and manual dissection as described by (*73*). Brains used for CUT&Tag and ATACseq were dissected from animals fixed in 2% formaldehyde to better preserve the chromatin and avoid disruption of histones.

Planarian intestinal cells were isolated using a protocol adapted from (*36*). Briefly, 125 μl of basic MACS beads (Miltenyi Biotec) were fed to large animals. After 48 hours, animals were dissociated as described previously and above (*10*), with the addition of 2 mM EDTA to the dissociation buffer (CMFB-E). The cell suspension was flowed through a MACS LS column (Miltenyi Biotec) and washed with CMFB-E three times. After removal from the magnet, intestinal cells were eluted using 3 × 3 ml ice-cold CMFB-E. Cells were inspected and counted using a hemocytometer.

Planarian epidermis used for RNAseq was isolated as described by (*74*), with the alteration that 7.8% ammonium thiocyanate (Sigma-Aldrich) in PBS was used. Epidermal cells used for ATACseq and CUT&Tag were isolated from dissociated cells based on the adhesive nature of these cells using MACS LS columns at low EDTA concentration (0.5 mM).

The purity of all isolated tissues was confirmed by qPCR for established planarian tissue markers (for primers, see table S5).

### RNAseq library preparation

RNAseq of tissue isolations was applied to determine the gene transcripts enriched in each of the tissues. RNAseq libraries were prepared using the Illumina TruSeq system according to the manufacturer’s instructions and were sequenced on an Illumina NovaSeq platform. Library preparation for each sample type was executed in triplicate.

### ATACseq library preparation

To identify which genomic regions are accessible in the cell of each of the tissue isolations, we performed ATACseq as described previously in (*30*). Tn5 transposase was purified using the construct pTXB1-Tn5 (Addgene #60240) (*75*). Transposase activity was verified by cleavage assay using linearized plasmid. ATACseq libraries were prepared according to the OMNI-ATAC protocol as described in (*76*). Briefly, samples consisting of 100,000 neoblasts, epidermal cells, intestinal cells, or cells from four brains were permeabilized in resuspension buffer (10 mM tris-HCl, 10 mM NaCl, 3 mM MgCl_2_) with detergents (0.1% NP-40, 0.1% Tween 20, 0.01% digitonin), washed, and incubated in resuspension buffer with Tn5 for 30 min at 37°C. Tagmented DNA was purified, amplified, and submitted for sequencing. Library preparation for each sample type was executed in triplicate.

### CUT&Tag library preparation

We used CUT&Tag (*17*) in each of the tissue isolations to identify which regions of the genome are marked by trimethylation of histone 3 on lysine 4 (H3K4me3), which correlates with promoter activity. Libraries were prepared as previously described in (*30*). Briefly, samples consisting of 100,000 neoblasts, epidermal cells, intestinal cells, or cells from four brains were permeabilized in NE1 buffer (20 mM Hepes-KOH, pH 7.9, 10 mM KCl, 0.5 mM spermidine, 0.1% Triton X-100, and 20% glycerol) and fixed in 0.1% formaldehyde for 2 min at 20°C. Samples were incubated with primary antibody rabbit anti-H3K4me3 (Millipore, 07-473) at 4°C overnight. After washing, samples were incubated with secondary antibody guinea pig anti-rabbit (Novusbio) for 1 hour at 20°C, followed by binding of pA-Tn5 for 1 hour at 20°C, and tagmentation in the presence of 10 mM MgCl_2_ for 1 hour at 37°C. Tagmented DNA was purified, amplified, and submitted for sequencing. Library preparation for each sample type was executed in triplicate.

### ATACseq and CUT&Tag data processing

In addition to new datasets generated for this study, we reanalyzed our previously published datasets from neoblasts, epidermis, and brain (*30*). Paired-end reads were filtered and trimmed using fastp (version 0.21.0 with --length_required 20 --average_qual 20) (*77*). Sequencing datasets were aligned against the *S. mediterranea* genome using bowtie2 (-X 2000 --very-sensitive) (*78*). The mapped reads were filtered based on mapping quality using samtools (-q 20) (*79*), and duplicates were removed using Picard. CUT&Tag and ATACseq peaks are called using MACS2 (*80*), using the following parameters: -g 7.63e8 --nomodel --extsize 200 --shift -100 --nolambda --call-summits. Peaks between replicates were considered conserved if the peak was present in all three replicates and if −log(*P* value) > 4. Peaks were then assigned to genomic features using BEDTools (*81*). deepTools (version 3.3) was used to obtain bw files and make profile plots (*82*).

To detect the region of maximum signal intensity of the ATACseq over the TSS (2 kb upstream of the TSS), BEDTools was used to overlap TSS and ATAC signal, and the region with maximum value was selected [>10 reads per kilobase per million (RPKM)].

Nucleosome occupancy analysis was carried out using NucleoATAC (version 0.3.4, default parameters) (*83*) over the TSS region ±1 kb. This script makes use of the fact that Tn5 insertions are unlikely to occur in the sequence that is bound by a nucleosome, but can occur on either side of it. It thus uses the lack of Tn5 cuts in combination with the fragment size of ATACseq reads to determine nucleosome enrichment relative to the TSS. Metaplots of the occ.bedgraph file (containing the nucleosome occupancy scores obtained from nucleoATAC) were plotted using deepTools.

In addition to data generated in this study, we reprocessed *S. mediterranea* ATACseq data (fig. S5, PRJNA633618) and *H. miamia* ATACseq data from previous studies (PRJNA512373, PRJNA832235, PRJNA515075) (*30*, *32*), which confirmed our findings (fig. S6). Our H3K4me3 CUT&Tag data were benchmarked against published *S. mediterranea* ChIPseq data (fig. S1E, GSE74169) (*18*). H3K4me3 ChIPseq was also previously performed in (*19*) with similar results.

### RNAseq analysis and identification of specific gene sets

Reads were filtered and trimmed using fastp (version 0.21.0 with --length_required 20 --average_qual 20) (*77*). Datasets were aligned against the *S. mediterranea* genome using STAR (version 2.7.2a) (--outFilterMismatchNmax 2 --alignIntronMax 15000 --alignMatesGapMax 15000 --outFilterMultimapNmax 100 --winAnchorMultimapNmax 100) (*84*). featureCounts (version 1.6.4, parameters: -M -C -O) (*85*) was used to count the reads/fragments over the gene annotations. Differential expression analysis was performed using DESeq2 version 1.26 (*86*) on raw read counts to obtain normalized fold changes (FCs) and *P*_adj_ values for each gene using the statistical models included in the DESeq2 package. Heatmaps were drawn with python and seaborn.

Pairwise comparisons were made between all the tissues (epidermis, brain, intestine, pharynx) and the neoblasts to define the specifically enriched gene clusters. Genes were considered differentially expressed only if they showed log_2_FC > 1 or log_2_FC < −1 and *P*_adj_ < 0.05. TPM values were also calculated for annotated transcripts. If a gene was identified as up-regulated in the neoblast isolations (more than twofold higher expression with an adjusted *P* value of less than 0.05) compared to each of the tissue isolations, it was defined as neoblast specific. If a gene was up-regulated in one specific tissue isolation compared to each of the other tissues and the neoblasts, it was defined as tissue specific. If the gene was expressed in all the samples and all *P*_adj_ < 0.05, the gene was classified as constitutive. The remaining genes were excluded from further analysis. Genes with TPM > 10 were classified as expressed; genes with TPM < 10 were marked as weakly expressed.

### Single-cell RNAseq analysis

To verify the various tissue isolations, we reprocessed single-cell RNAseq data available from Wurtzel *et al.* (*16*) to compare expression profiles of the tissues to the cell types identified from single-cell analysis. Reads were filtered and trimmed using fastp (version 0.21.0 with --length_required 20 --average_qual 20) (*77*). Datasets were aligned against the *S. mediterranea* genome using Salmon (*87*), using the Unigene annotation (*69*), followed by Seurat for cluster detection (*88*). Samples expressing less than 1000 or more than 20,000 genes were removed. The remaining cells were clustered into 14 distinct major clusters using a graph-based clustering approach and were visualized by applying the embedded t-distributed stochastic neighbor embedding (t-SNE) capabilities of Seurat. The same processing was used for *H. miamia* data (PRJNA889328), using the published *Hofstenia* transcriptome (*32*).

### GO term analysis

To verify the tissue isolations and the identification procedure for the specific gene sets, we used GO analysis to assess whether the identified enriched transcripts in each isolation matched expectations for that tissue. Analysis of GO terms in nonannotated model systems is problematic, and this analysis was therefore only used to verify global gene enrichment patterns and is not used to draw any conclusions about the presence or absence of individual genes or gene classes. GO terms were associated with each transcript by adopting the terms from the best human homolog as determined by blastx if the *e* value was below 1 × 10^−10^. Transcripts that did not meet the *e*-value threshold were excluded from further analysis. For each tissue, the best human homologs of the transcripts enriched in the tissue were compared to the total set of best human homologs of the planarian transcriptome using DAVID (*89*) for enrichment analysis.

### Hi-C analysis

Data from Guo *et al.* (*50*) (SRR14597012) were mapped to the most recent *S. mediterranea* genome assembly and further cleaned up to remove duplicates and self-ligations using the Juicer (v1.6) pipeline with the default parameters (*90*). The current assembly of the planarian genome contains regions that are not covered by Hi-C reads. To avoid artifacts in the computation of eigenvectors introduced by these uncovered regions, we followed a strategy previously introduced by Rowley *et al.* (*91*). We generated a custom script that removed the rows and columns with low coverage (“white strips”), then recomputed the eigenvector from the Pearson correlation matrix of Hi-C data, and finally reallocated the correct coordinates to the values obtained. Compartments were called at 50-kb resolution using the KR normalized reads (*49*) and were visualized using Juicebox (*90*).

### Motif analysis

To analyze enrichment of known TF motifs (table S2), FIMO (*92*) was used to scan the region 2 kb upstream of the TSS for each gene. To identify de novo motif enrichment in tissue-specific, neoblast-specific, and constitutive ATACseq peaks, HOMER (*93*) findMotifs.pl was applied. We used regions of 100 nt centered on the ATAC peak center including 412 regions for intestine, 362 for epidermis, 169 regions for neoblast, and 106 regions for constitutive genes that were accessible in at least one of our tissues of interest and located over the promoter of the constitutive genes. The background data were determined using the HOMER script scrambleFasta.pl for each cluster of genes. Similar results were obtained using the MEME software suite (*94*, *95*).

The putative planarian Initiator sequence (table S3) was discovered over the TSS (±40 nt) by HOMER findMotifs.pl as well as by STREME (*95*). The presence of this motif and the presence of the TATA box at the TSS of different gene clusters were analyzed by HOMER findMotifs.pl (-find parameter). Periodicity of dinucleotides over the regions surrounding the TSS was scanned using the periodicDNA R package (*96*), but none was detected.

### Image acquisition and analysis

To assure unbiased and consistent quantification of labeled cells, the acquired images were analyzed automatically using a method adapted from the previously published ImageJ plugin NODeJ (*61*). Briefly, we performed a Laplacian convolution of size *n* on the images to enhance the contrast of the objects of interest and allow their detection using a threshold computed by a factor *f* on the image. The values for parameters *n* and *f* need to be adjusted for each staining type due to differences in the distribution of the signal intensity. Identified objects were then extracted for further automated analysis as described below.

For the analysis of homeostatic animals, images were acquired on a Zeiss LSM800 confocal microscope. Control and experimental RNAi-treated animals were imaged at ×20 magnification (voxel calibration: *x*: 1.38 μm, *y*: 1.38 μm, and *z*: 7 μm), at comparable anatomical position (around the pharynx). Objects were detected by automated segmentation in 2D using the following values for parameters *n* and *f*: 4′,6-diamidino-2-phenylindole (DAPI) (*n* = 2 and *f* = 1) and *smedwi-1* fluorescence in situ hybridization (FISH) (*n* = 3 and *f* = 2). Detected *smedwi-1* objects were counted and normalized to the number of DAPI-positive nuclei per image to control for differences in cell number between images.

For the analysis of neoblast expansion after irradiation, images were taken on a Zeiss LSM800 confocal microscope at ×10 magnification (voxel calibration: *x*: 2.76 μm, *y*: 2.76 μm, *z*: 7 μm). Objects were analyzed in 3D with parameter settings *n* = 3 and *f* = 1.5. Centroids were computed for each detected object, and the Euclidean distance was computed between all the centroids. If two centroids were at a distance less than 50 μm, those two cells were defined as part of the same cluster.

### Statistical analysis

Levels of significance as shown in Figs. 1H, 2B, 3K, 4D, and 5 (G and H) were calculated using a Wilcoxon test as implemented in ggpubR in the R software environment (version 4.1.2). Statistical analysis of qPCR data shown in Fig. 5 (B and C) and figs. S7C and S14 was calculated with a two-tailed Student’s *t* test using the Prism software package. Correlation coefficients shown in fig. S4 were determined by Pearson correlation performed with deepTools (version 3.5.1). Analysis of genome-wide data was carried out as described above.

## References

[1] S. Masui, Y. Nakatake, Y. Toyooka, D. Shimosato, R. Yagi, K. Takahashi, H. Okochi, A. Okuda, R. Matoba, A. A. Sharov, M. S. H. Ko, H. Niwa, Pluripotency governed by Sox2 via regulation of Oct3/4 expression in mouse embryonic stem cells. Nat. Cell Biol. 9, 625–635 (2007). 17515932 10.1038/ncb1589

[2] J. Halliwell, I. Barbaric, P. W. Andrews, Acquired genetic changes in human pluripotent stem cells: Origins and consequences. Nat. Rev. Mol. Cell Biol. 21, 715–728 (2020). 32968234 10.1038/s41580-020-00292-z

[3] S. Bar, N. Benvenisty, Epigenetic aberrations in human pluripotent stem cells. EMBO J. 38, e101033 (2019). 31088843 10.15252/embj.2018101033 PMC6576196

[4] F. T. Merkle, S. Ghosh, N. Kamitaki, J. Mitchell, Y. Avior, C. Mello, S. Kashin, S. Mekhoubad, D. Ilic, M. Charlton, G. Saphier, R. E. Handsaker, G. Genovese, S. Bar, N. Benvenisty, S. A. McCarroll, K. Eggan, Human pluripotent stem cells recurrently acquire and expand dominant negative P53 mutations. Nature 545, 229–233 (2017). 28445466 10.1038/nature22312 PMC5427175

[5] D. E. Wagner, I. E. Wang, P. W. Reddien, Clonogenic neoblasts are pluripotent adult stem cells that underlie planarian regeneration. Science 332, 811–816 (2011). 21566185 10.1126/science.1203983 PMC3338249

[6] P. W. Reddien, N. J. Oviedo, J. R. Jennings, J. C. Jenkin, A. Sanchez Alvarado, SMEDWI-2 is a PIWI-like protein that regulates planarian stem cells. Science 310, 1327–1330 (2005). 16311336 10.1126/science.1116110

[7] T. Guo, A. H. Peters, P. A. Newmark, A Bruno-like gene is required for stem cell maintenance in planarians. Dev. Cell 11, 159–169 (2006). 16890156 10.1016/j.devcel.2006.06.004

[8] J. Solana, D. Kao, Y. Mihaylova, F. Jaber-Hijazi, S. Malla, R. Wilson, A. Aboobaker, Defining the molecular profile of planarian pluripotent stem cells using a combinatorial RNAseq, RNA interference and irradiation approach. Genome Biol. 13, R19 (2012). 22439894 10.1186/gb-2012-13-3-r19 PMC3439970

[9] P. Onal, D. Grün, C. Adamidi, A. Rybak, J. Solana, G. Mastrobuoni, Y. Wang, H.-P. Rahn, W. Chen, S. Kempa, U. Ziebold, N. Rajewsky, Gene expression of pluripotency determinants is conserved between mammalian and planarian stem cells. EMBO J. 31, 2755–2769 (2012). 22543868 10.1038/emboj.2012.110 PMC3380209

[10] J. C. van Wolfswinkel, D. E. Wagner, P. W. Reddien, Single-cell analysis reveals functionally distinct classes within the planarian stem cell compartment. Cell Stem Cell 15, 326–339 (2014). 25017721 10.1016/j.stem.2014.06.007 PMC4171737

[11] A. A. Raz, O. Wurtzel, P. W. Reddien, Planarian stem cells specify fate yet retain potency during the cell cycle. Cell Stem Cell 28, 1307–1322.e5 (2021). 33882291 10.1016/j.stem.2021.03.021 PMC8254784

[12] M. L. Scimone, K. M. Kravarik, S. W. Lapan, P. W. Reddien, Neoblast specialization in regeneration of the planarian Schmidtea mediterranea. Stem Cell Rep. 3, 339–352 (2014). 10.1016/j.stemcr.2014.06.001 PMC4176530 25254346

[13] A. Zeng, H. Li, L. Guo, X. Gao, S. McKinney, Y. Wang, Z. Yu, J. Park, C. Semerad, E. Ross, L. C. Cheng, E. Davies, K. Lei, W. Wang, A. Perera, K. Hall, A. Peak, A. Box, A. Sánchez Alvarado, Prospectively isolated tetraspanin^+^ neoblasts are adult pluripotent stem cells underlying planaria regeneration. Cell 173, 1593–1608.e20 (2018). 29906446 10.1016/j.cell.2018.05.006 PMC9359418

[14] C. T. Fincher, O. Wurtzel, T. de Hoog, K. M. Kravarik, P. W. Reddien, Cell type transcriptome atlas for the planarian Schmidtea mediterranea. Science 360, eaaq1736 (2018). 29674431 10.1126/science.aaq1736 PMC6563842

[15] M. Plass, J. Solana, F. A. Wolf, S. Ayoub, A. Misios, P. Glažar, B. Obermayer, F. J. Theis, C. Kocks, N. Rajewsky, Cell type atlas and lineage tree of a whole complex animal by single-cell transcriptomics. Science 360, eaaq1723 (2018). 29674432 10.1126/science.aaq1723

[16] O. Wurtzel, L. E. Cote, A. Poirier, R. Satija, A. Regev, P. W. Reddien, A generic and cell-type-specific wound response precedes regeneration in planarians. Dev. Cell 35, 632–645 (2015). 26651295 10.1016/j.devcel.2015.11.004 PMC4817857

[17] H. S. Kaya-Okur, S. J. Wu, C. A. Codomo, E. S. Pledger, T. D. Bryson, J. G. Henikoff, K. Ahmad, S. Henikoff, CUT&Tag for efficient epigenomic profiling of small samples and single cells. Nat. Commun. 10, 1930 (2019). 31036827 10.1038/s41467-019-09982-5 PMC6488672

[18] E. M. Duncan, A. D. Chitsazan, C. W. Seidel, A. S. Alvarado, Set1 and MLL1/2 target distinct sets of functionally different genomic loci in vivo. Cell Rep. 13, 2741–2755 (2015). 26711341 10.1016/j.celrep.2015.11.059 PMC4707048

[19] A. Dattani, D. Kao, Y. Mihaylova, P. Abnave, S. Hughes, A. Lai, S. Sahu, A. A. Aboobaker, Epigenetic analyses of planarian stem cells demonstrate conservation of bivalent histone modifications in animal stem cells. Genome Res. 28, 1543–1554 (2018). 30143598 10.1101/gr.239848.118 PMC6169894

[20] J. D. Buenrostro, P. G. Giresi, L. C. Zaba, H. Y. Chang, W. J. Greenleaf, Transposition of native chromatin for fast and sensitive epigenomic profiling of open chromatin, DNA-binding proteins and nucleosome position. Nat. Methods 10, 1213–1218 (2013). 24097267 10.1038/nmeth.2688 PMC3959825

[21] G. C. Yuan, Y. J. Liu, M. F. Dion, M. D. Slack, L. F. Wu, S. J. Altschuler, O. J. Rando, Genome-scale identification of nucleosome positions in S. cerevisiae. Science 309, 626–630 (2005). 15961632 10.1126/science.1112178

[22] T. N. Mavrich, C. Jiang, I. P. Ioshikhes, X. Li, B. J. Venters, S. J. Zanton, L. P. Tomsho, J. Qi, R. L. Glaser, S. C. Schuster, D. S. Gilmour, I. Albert, B. F. Pugh, Nucleosome organization in the Drosophila genome. Nature 453, 358–362 (2008). 18408708 10.1038/nature06929 PMC2735122

[23] A. Valouev, J. Ichikawa, T. Tonthat, J. Stuart, S. Ranade, H. Peckham, K. Zeng, J. A. Malek, G. Costa, K. McKernan, A. Sidow, A. Fire, S. M. Johnson, A high-resolution, nucleosome position map of C. elegans reveals a lack of universal sequence-dictated positioning. Genome Res. 18, 1051–1063 (2008). 18477713 10.1101/gr.076463.108 PMC2493394

[24] V. R. Yella, M. Bansal, DNA structural features of eukaryotic TATA-containing and TATA-less promoters. FEBS Open Bio 7, 324–334 (2017). 10.1002/2211-5463.12166 PMC5337902 28286728

[25] Y. Field, N. Kaplan, Y. Fondufe-Mittendorf, I. K. Moore, E. Sharon, Y. Lubling, J. Widom, E. Segal, Distinct modes of regulation by chromatin encoded through nucleosome positioning signals. PLOS Comput. Biol. 4, e1000216 (2008). 18989395 10.1371/journal.pcbi.1000216 PMC2570626

[26] I. Tirosh, N. Barkai, Two strategies for gene regulation by promoter nucleosomes. Genome Res. 18, 1084–1091 (2008). 18448704 10.1101/gr.076059.108 PMC2493397

[27] E. A. Rach, D. R. Winter, A. M. Benjamin, D. L. Corcoran, T. Ni, J. Zhu, U. Ohler, Transcription initiation patterns indicate divergent strategies for gene regulation at the chromatin level. PLOS Genet. 7, e1001274 (2011). 21249180 10.1371/journal.pgen.1001274 PMC3020932

[28] P. Carninci, A. Sandelin, B. Lenhard, S. Katayama, K. Shimokawa, J. Ponjavic, C. A. M. Semple, M. S. Taylor, P. G. Engström, M. C. Frith, A. R. R. Forrest, W. B. Alkema, S. L. Tan, C. Plessy, R. Kodzius, T. Ravasi, T. Kasukawa, S. Fukuda, M. Kanamori-Katayama, Y. Kitazume, H. Kawaji, C. Kai, M. Nakamura, H. Konno, K. Nakano, S. Mottagui-Tabar, P. Arner, A. Chesi, S. Gustincich, F. Persichetti, H. Suzuki, S. M. Grimmond, C. A. Wells, V. Orlando, C. Wahlestedt, E. T. Liu, M. Harbers, J. Kawai, V. B. Bajic, D. A. Hume, Y. Hayashizaki, Genome-wide analysis of mammalian promoter architecture and evolution. Nat. Genet. 38, 626–635 (2006). 16645617 10.1038/ng1789

[29] J. Serizay, Y. Dong, J. Jänes, M. Chesney, C. Cerrato, J. Ahringer, Distinctive regulatory architectures of germline-active and somatic genes in C. elegans. Genome Res. 30, 1752–1765 (2020). 33093068 10.1101/gr.265934.120 PMC7706728

[30] D. Li, D. H. Taylor, J. C. van Wolfswinkel, PIWI-mediated control of tissue-specific transposons is essential for somatic cell differentiation. Cell Rep. 37, 109776 (2021). 34610311 10.1016/j.celrep.2021.109776 PMC8532177

[31] J. Neiro, D. Sridhar, A. Dattani, A. Aboobaker, Identification of putative enhancer-like elements predicts regulatory networks active in planarian adult stem cells. eLife 11, e79675 (2022). 35997250 10.7554/eLife.79675 PMC9522251

[32] A. R. Gehrke, E. Neverett, Y. J. Luo, A. Brandt, L. Ricci, R. E. Hulett, A. Gompers, J. G. Ruby, D. S. Rokhsar, P. W. Reddien, M. Srivastava, Acoel genome reveals the regulatory landscape of whole-body regeneration. Science 363, eaau6173 (2019). 30872491 10.1126/science.aau6173

[33] J. O. Kimura, D. M. Bolanos, L. Ricci, M. Srivastava, Embryonic origins of adult pluripotent stem cells. Cell 185, 4756–4769.e13 (2022). 36493754 10.1016/j.cell.2022.11.008 PMC9761687

[34] T. W. Whitfield, J. Wang, P. J. Collins, E. C. Partridge, S. Aldred, N. D. Trinklein, R. M. Myers, Z. Weng, Functional analysis of transcription factor binding sites in human promoters. Genome Biol. 13, R50 (2012). 22951020 10.1186/gb-2012-13-9-r50 PMC3491394

[35] B. Ren, F. Robert, J. J. Wyrick, O. Aparicio, E. G. Jennings, I. Simon, J. Zeitlinger, J. Schreiber, N. Hannett, E. Kanin, T. L. Volkert, C. J. Wilson, S. P. Bell, R. A. Young, Genome-wide location and function of DNA binding proteins. Science 290, 2306–2309 (2000). 11125145 10.1126/science.290.5500.2306

[36] D. J. Forsthoefel, N. P. James, D. J. Escobar, J. M. Stary, A. P. Vieira, F. A. Waters, P. A. Newmark, An RNAi screen reveals intestinal regulators of branching morphogenesis, differentiation, and stem cell proliferation in planarians. Dev. Cell 23, 691–704 (2012). 23079596 10.1016/j.devcel.2012.09.008 PMC3521571

[37] L. C. Cheng, K. C. Tu, C. W. Seidel, S. M. C. Robb, F. Guo, A. Sánchez Alvarado, Cellular, ultrastructural and molecular analyses of epidermal cell development in the planarian Schmidtea mediterranea. Dev. Biol. 433, 357–373 (2018). 29100657 10.1016/j.ydbio.2017.08.030 PMC5750087

[38] K. Struhl, Naturally occurring poly(dA-dT) sequences are upstream promoter elements for constitutive transcription in yeast. Proc. Natl. Acad. Sci. U.S.A. 82, 8419–8423 (1985). 3909145 10.1073/pnas.82.24.8419 PMC390927

[39] V. Iyer, K. Struhl, Poly(dA:dT), a ubiquitous promoter element that stimulates transcription via its intrinsic DNA structure. EMBO J. 14, 2570–2579 (1995). 7781610 10.1002/j.1460-2075.1995.tb07255.x PMC398371

[40] K. J. Dechering, K. Cuelenaere, R. N. Konings, J. A. Leunissen, Distinct frequency-distributions of homopolymeric DNA tracts in different genomes. Nucleic Acids Res. 26, 4056–4062 (1998). 9705519 10.1093/nar/26.17.4056 PMC147789

[41] K. A. Koch, D. J. Thiele, Functional analysis of a homopolymeric (dA-dT) element that provides nucleosomal access to yeast and mammalian transcription factors. J. Biol. Chem. 274, 23752–23760 (1999). 10446135 10.1074/jbc.274.34.23752

[42] H. Ellegren, Microsatellites: Simple sequences with complex evolution. Nat. Rev. Genet. 5, 435–445 (2004). 15153996 10.1038/nrg1348

[43] J. Quilez, A. Guilmatre, P. Garg, G. Highnam, M. Gymrek, Y. Erlich, R. S. Joshi, D. Mittelman, A. J. Sharp, Polymorphic tandem repeats within gene promoters act as modifiers of gene expression and DNA methylation in humans. Nucleic Acids Res. 44, 3750–3762 (2016). 27060133 10.1093/nar/gkw219 PMC4857002

[44] M. Gymrek, T. Willems, A. Guilmatre, H. Zeng, B. Markus, S. Georgiev, M. J. Daly, A. L. Price, J. K. Pritchard, A. J. Sharp, Y. Erlich, Abundant contribution of short tandem repeats to gene expression variation in humans. Nat. Genet. 48, 22–29 (2016). 26642241 10.1038/ng.3461 PMC4909355

[45] H. Y. Chen, S. L. Ma, W. Huang, L. Ji, V. H. K. Leung, H. Jiang, X. Yao, N. L. S. Tang, The mechanism of transactivation regulation due to polymorphic short tandem repeats (STRs) using IGF1 promoter as a model. Sci. Rep. 6, 38225 (2016). 27910883 10.1038/srep38225 PMC5133613

[46] M. A. Grohme, S. Schloissnig, A. Rozanski, M. Pippel, G. R. Young, S. Winkler, H. Brandl, I. Henry, A. Dahl, S. Powell, M. Hiller, E. Myers, J. C. Rink, The genome of Schmidtea mediterranea and the evolution of core cellular mechanisms. Nature 554, 56–61 (2018). 29364871 10.1038/nature25473 PMC5797480

[47] E. Lieberman-Aiden, N. L. van Berkum, L. Williams, M. Imakaev, T. Ragoczy, A. Telling, I. Amit, B. R. Lajoie, P. J. Sabo, M. O. Dorschner, R. Sandstrom, B. Bernstein, M. A. Bender, M. Groudine, A. Gnirke, J. Stamatoyannopoulos, L. A. Mirny, E. S. Lander, J. Dekker, Comprehensive mapping of long-range interactions reveals folding principles of the human genome. Science 326, 289–293 (2009). 19815776 10.1126/science.1181369 PMC2858594

[48] T. Sexton, E. Yaffe, E. Kenigsberg, F. Bantignies, B. Leblanc, M. Hoichman, H. Parrinello, A. Tanay, G. Cavalli, Three-dimensional folding and functional organization principles of the Drosophila genome. Cell 148, 458–472 (2012). 22265598 10.1016/j.cell.2012.01.010

[49] S. S. P. Rao, M. H. Huntley, N. C. Durand, E. K. Stamenova, I. D. Bochkov, J. T. Robinson, A. L. Sanborn, I. Machol, A. D. Omer, E. S. Lander, E. L. Aiden, A 3D map of the human genome at kilobase resolution reveals principles of chromatin looping. Cell 159, 1665–1680 (2014). 25497547 10.1016/j.cell.2014.11.021 PMC5635824

[50] L. Guo, J. S. Bloom, D. Dols-Serrate, J. Boocock, E. Ben-David, O. T. Schubert, K. Kozuma, K. Ho, E. Warda, C. Chui, Y. Wei, D. Leighton, T. Lemus Vergara, M. Riutort, A. Sánchez Alvarado, L. Kruglyak, Island-specific evolution of a sex-primed autosome in a sexual planarian. Nature 606, 329–334 (2022). 35650439 10.1038/s41586-022-04757-3 PMC9177419

[51] H. L. Harris, H. Gu, M. Olshansky, A. Wang, I. Farabella, Y. Eliaz, A. Kalluchi, A. Krishna, M. Jacobs, G. Cauer, M. Pham, S. S. P. Rao, O. Dudchenko, A. Omer, K. Mohajeri, S. Kim, M. H. Nichols, E. S. Davis, D. Gkountaroulis, D. Udupa, A. P. Aiden, V. G. Corces, D. H. Phanstiel, W. S. Noble, G. Nir, M. di Pierro, J. S. Seo, M. E. Talkowski, E. L. Aiden, M. J. Rowley, Chromatin alternates between A and B compartments at kilobase scale for subgenic organization. Nat. Commun. 14, 3303 (2023). 37280210 10.1038/s41467-023-38429-1 PMC10244318

[52] H. C. Nelson, J. T. Finch, B. F. Luisi, A. Klug, The structure of an oligo(dA).oligo(dT) tract and its biological implications. Nature 330, 221–226 (1987). 3670410 10.1038/330221a0

[53] B. Suter, G. Schnappauf, F. Thoma, Poly(dA.dT) sequences exist as rigid DNA structures in nucleosome-free yeast promoters *in vivo*. Nucleic Acids Res. 28, 4083–4089 (2000). 11058103 10.1093/nar/28.21.4083 PMC113125

[54] Y. Bao, C. L. White, K. Luger, Nucleosome core particles containing a poly(dA.dT) sequence element exhibit a locally distorted DNA structure. J. Mol. Biol. 361, 617–624 (2006). 16860337 10.1016/j.jmb.2006.06.051

[55] J. D. Anderson, J. Widom, Poly(dA-dT) promoter elements increase the equilibrium accessibility of nucleosomal DNA target sites. Mol. Cell. Biol. 21, 3830–3839 (2001). 11340174 10.1128/MCB.21.11.3830-3839.2001 PMC87046

[56] E. Segal, J. Widom, Poly(dA:dT) tracts: Major determinants of nucleosome organization. Curr. Opin. Struct. Biol. 19, 65–71 (2009). 19208466 10.1016/j.sbi.2009.01.004 PMC2673466

[57] Y. Lorch, B. Maier-Davis, R. D. Kornberg, Role of DNA sequence in chromatin remodeling and the formation of nucleosome-free regions. Genes Dev. 28, 2492–2497 (2014). 25403179 10.1101/gad.250704.114 PMC4233242

[58] S. Kubik, M. J. Bruzzone, D. Challal, R. Dreos, S. Mattarocci, P. Bucher, D. Libri, D. Shore, Opposing chromatin remodelers control transcription initiation frequency and start site selection. Nat. Struct. Mol. Biol. 26, 744–754 (2019). 31384063 10.1038/s41594-019-0273-3

[59] T. Trost, J. Haines, A. Dillon, B. Mersman, M. Robbins, P. Thomas, A. Hubert, Characterizing the role of SWI/SNF-related chromatin remodeling complexes in planarian regeneration and stem cell function. Stem Cell Res. 32, 91–103 (2018). 30237141 10.1016/j.scr.2018.09.004

[60] M. L. Scimone, J. Meisel, P. W. Reddien, The Mi-2-like Smed-CHD4 gene is required for stem cell differentiation in the planarian Schmidtea mediterranea. Development 137, 1231–1241 (2010). 20223763 10.1242/dev.042051 PMC2847463

[61] T. Dubos, A. Poulet, G. Thomson, E. Péry, F. Chausse, C. Tatout, S. Desset, J. C. van Wolfswinkel, Y. Jacob, NODeJ: An ImageJ plugin for 3D segmentation of nuclear objects. BMC Bioinformatics 23, 216 (2022). 35668354 10.1186/s12859-022-04743-6 PMC9169307

[62] W. Sukparangsi, E. Morganti, M. Lowndes, H. Mayeur, M. Weisser, F. Hammachi, H. Peradziryi, F. Roske, J. Hölzenspies, A. Livigni, B. G. Godard, F. Sugahara, S. Kuratani, G. Montoya, S. R. Frankenberg, S. Mazan, J. M. Brickman, Evolutionary origin of vertebrate OCT4/POU5 functions in supporting pluripotency. Nat. Commun. 13, 5537 (2022). 36130934 10.1038/s41467-022-32481-z PMC9492771

[63] D. A. Gold, R. D. Gates, D. K. Jacobs, The early expansion and evolutionary dynamics of POU class genes. Mol. Biol. Evol. 31, 3136–3147 (2014). 25261405 10.1093/molbev/msu243 PMC4245813

[64] H. W. King, R. J. Klose, The pioneer factor OCT4 requires the chromatin remodeller BRG1 to support gene regulatory element function in mouse embryonic stem cells. eLife 6, e22631 (2017). 28287392 10.7554/eLife.22631 PMC5400504

[65] E. T. Friman, C. Deluz, A. C. A. Meireles-Filho, S. Govindan, V. Gardeux, B. Deplancke, D. M. Suter, Dynamic regulation of chromatin accessibility by pluripotency transcription factors across the cell cycle. eLife 8, e50087 (2019). 31794382 10.7554/eLife.50087 PMC6890464

[66] M. L. Scimone, O. Wurtzel, K. Malecek, C. T. Fincher, I. M. Oderberg, K. M. Kravarik, P. W. Reddien, foxF-1 controls specification of non-body wall muscle and phagocytic cells in planarians. Curr. Biol. 28, 3787–3801.e6 (2018). 30471994 10.1016/j.cub.2018.10.030 PMC6411049

[67] P. A. Newmark, A. Sanchez Alvarado, Bromodeoxyuridine specifically labels the regenerative stem cells of planarians. Dev. Biol. 220, 142–153 (2000). 10753506 10.1006/dbio.2000.9645

[68] S. Y. Liu, C. Selck, B. Friedrich, R. Lutz, M. Vila-Farré, A. Dahl, H. Brandl, N. Lakshmanaperumal, I. Henry, J. C. Rink, Reactivating head regrowth in a regeneration-deficient planarian species. Nature 500, 81–84 (2013). 23883932 10.1038/nature12414

[69] S. M. Robb, K. Gotting, E. Ross, A. S. Alvarado, SmedGD 2.0: The Schmidtea mediterranea genome database. Genesis 53, 535–546 (2015). 26138588 10.1002/dvg.22872 PMC4867232

[70] B. J. Pearson, G. T. Eisenhoffer, K. A. Gurley, J. C. Rink, D. E. Miller, A. Sánchez Alvarado, Formaldehyde-based whole-mount in situ hybridization method for planarians. Dev. Dyn. 238, 443–450 (2009). 19161223 10.1002/dvdy.21849 PMC2640425

[71] R. S. King, P. A. Newmark, In situ hybridization protocol for enhanced detection of gene expression in the planarian *Schmidtea mediterranea*. BMC Dev. Biol. 13, 8 (2013). 23497040 10.1186/1471-213X-13-8 PMC3610298

[72] T. Hayashi, M. Asami, S. Higuchi, N. Shibata, K. Agata, Isolation of planarian X-ray-sensitive stem cells by fluorescence-activated cell sorting. Dev. Growth Differ. 48, 371–380 (2006). 16872450 10.1111/j.1440-169X.2006.00876.x

[73] I. E. Wang, S. W. Lapan, M. L. Scimone, T. R. Clandinin, P. W. Reddien, Hedgehog signaling regulates gene expression in planarian glia. eLife 5, e16996 (2016). 27612382 10.7554/eLife.16996 PMC5055395

[74] O. Wurtzel, I. M. Oderberg, P. W. Reddien, Planarian epidermal stem cells respond to positional cues to promote cell-type diversity. Dev. Cell 40, 491–504.e5 (2017). 28292427 10.1016/j.devcel.2017.02.008 PMC5679284

[75] S. Picelli, O. R. Faridani, Å. K. Björklund, G. Winberg, S. Sagasser, R. Sandberg, Full-length RNA-seq from single cells using Smart-seq2. Nat. Protoc. 9, 171–181 (2014). 24385147 10.1038/nprot.2014.006

[76] M. R. Corces, A. E. Trevino, E. G. Hamilton, P. G. Greenside, N. A. Sinnott-Armstrong, S. Vesuna, A. T. Satpathy, A. J. Rubin, K. S. Montine, B. Wu, A. Kathiria, S. W. Cho, M. R. Mumbach, A. C. Carter, M. Kasowski, L. A. Orloff, V. I. Risca, A. Kundaje, P. A. Khavari, T. J. Montine, W. J. Greenleaf, H. Y. Chang, An improved ATAC-seq protocol reduces background and enables interrogation of frozen tissues. Nat. Methods 14, 959–962 (2017). 28846090 10.1038/nmeth.4396 PMC5623106

[77] S. Chen, Y. Zhou, Y. Chen, J. Gu, fastp: An ultra-fast all-in-one FASTQ preprocessor. Bioinformatics 34, i884–i890 (2018). 30423086 10.1093/bioinformatics/bty560 PMC6129281

[78] B. Langmead, S. L. Salzberg, Fast gapped-read alignment with Bowtie 2. Nat. Methods 9, 357–359 (2012). 22388286 10.1038/nmeth.1923 PMC3322381

[79] H. Li, B. Handsaker, A. Wysoker, T. Fennell, J. Ruan, N. Homer, G. Marth, G. Abecasis, R. Durbin; 1000 Genome Project Data Processing Subgroup, The sequence alignment/map format and SAMtools. Bioinformatics 25, 2078–2079 (2009). 19505943 10.1093/bioinformatics/btp352 PMC2723002

[80] Y. Zhang, T. Liu, C. A. Meyer, J. Eeckhoute, D. S. Johnson, B. E. Bernstein, C. Nusbaum, R. M. Myers, M. Brown, W. Li, X. S. Liu, Model-based analysis of ChIP-Seq (MACS). Genome Biol. 9, R137 (2008). 18798982 10.1186/gb-2008-9-9-r137 PMC2592715

[81] A. R. Quinlan, BEDTools: The swiss-army tool for genome feature analysis. Curr. Protoc. Bioinformatics 47, 11.12.1–11.12.34 (2014). 10.1002/0471250953.bi1112s47 PMC4213956 25199790

[82] F. Ramirez, F. Dundar, S. Diehl, B. A. Gruning, T. Manke, deepTools: A flexible platform for exploring deep-sequencing data. Nucleic Acids Res. 42, W187–W191 (2014). 24799436 10.1093/nar/gku365 PMC4086134

[83] A. N. Schep, J. D. Buenrostro, S. K. Denny, K. Schwartz, G. Sherlock, W. J. Greenleaf, Structured nucleosome fingerprints enable high-resolution mapping of chromatin architecture within regulatory regions. Genome Res. 25, 1757–1770 (2015). 26314830 10.1101/gr.192294.115 PMC4617971

[84] A. Dobin, C. A. Davis, F. Schlesinger, J. Drenkow, C. Zaleski, S. Jha, P. Batut, M. Chaisson, T. R. Gingeras, STAR: Ultrafast universal RNA-seq aligner. Bioinformatics 29, 15–21 (2013). 23104886 10.1093/bioinformatics/bts635 PMC3530905

[85] Y. Liao, G. K. Smyth, W. Shi, featureCounts: An efficient general purpose program for assigning sequence reads to genomic features. Bioinformatics 30, 923–930 (2014). 24227677 10.1093/bioinformatics/btt656

[86] M. I. Love, W. Huber, S. Anders, Moderated estimation of fold change and dispersion for RNA-seq data with DESeq2. Genome Biol. 15, 550 (2014). 25516281 10.1186/s13059-014-0550-8 PMC4302049

[87] R. Patro, G. Duggal, M. I. Love, R. A. Irizarry, C. Kingsford, Salmon provides fast and bias-aware quantification of transcript expression. Nat. Methods 14, 417–419 (2017). 28263959 10.1038/nmeth.4197 PMC5600148

[88] R. Satija, J. A. Farrell, D. Gennert, A. F. Schier, A. Regev, Spatial reconstruction of single-cell gene expression data. Nat. Biotechnol. 33, 495–502 (2015). 25867923 10.1038/nbt.3192 PMC4430369

[89] B. T. Sherman, M. Hao, J. Qiu, X. Jiao, M. W. Baseler, H. C. Lane, T. Imamichi, W. Chang, DAVID: A web server for functional enrichment analysis and functional annotation of gene lists (2021 update). Nucleic Acids Res. 50, W216–W221 (2022). 35325185 10.1093/nar/gkac194 PMC9252805

[90] N. C. Durand, M. S. Shamim, I. Machol, S. S. P. Rao, M. H. Huntley, E. S. Lander, E. L. Aiden, Juicer provides a one-click system for analyzing loop-resolution Hi-C experiments. Cell Syst. 3, 95–98 (2016). 27467249 10.1016/j.cels.2016.07.002 PMC5846465

[91] M. J. Rowley, X. Lyu, V. Rana, M. Ando-Kuri, R. Karns, G. Bosco, V. G. Corces, Condensin II counteracts cohesin and RNA polymerase II in the establishment of 3D chromatin organization. Cell Rep. 26, 2890–2903.e3 (2019). 30865881 10.1016/j.celrep.2019.01.116 PMC6424357

[92] C. E. Grant, T. L. Bailey, W. S. Noble, FIMO: Scanning for occurrences of a given motif. Bioinformatics 27, 1017–1018 (2011). 21330290 10.1093/bioinformatics/btr064 PMC3065696

[93] S. Heinz, C. Benner, N. Spann, E. Bertolino, Y. C. Lin, P. Laslo, J. X. Cheng, C. Murre, H. Singh, C. K. Glass, Simple combinations of lineage-determining transcription factors prime cis-regulatory elements required for macrophage and B cell identities. Mol. Cell 38, 576–589 (2010). 20513432 10.1016/j.molcel.2010.05.004 PMC2898526

[94] T. L. Bailey, N. Williams, C. Misleh, W. W. Li, MEME: Discovering and analyzing DNA and protein sequence motifs. Nucleic Acids Res. 34, W369–W373 (2006). 16845028 10.1093/nar/gkl198 PMC1538909

[95] T. L. Bailey, STREME: Accurate and versatile sequence motif discovery. Bioinformatics 37, 2834–2840 (2021). 33760053 10.1093/bioinformatics/btab203 PMC8479671

[96] J. Serizay, J. Ahringer, periodicDNA: An R/Bioconductor package to investigate k-mer periodicity in DNA. F1000Res. 10, 141 (2021). 33953908 10.12688/f1000research.51143.1 PMC8063535

[97] T. Paysan-Lafosse, M. Blum, S. Chuguransky, T. Grego, B. L. Pinto, G. A. Salazar, M. L. Bileschi, P. Bork, A. Bridge, L. Colwell, J. Gough, D. H. Haft, I. Letunić, A. Marchler-Bauer, H. Mi, D. A. Natale, C. A. Orengo, A. P. Pandurangan, C. Rivoire, C. J. A. Sigrist, I. Sillitoe, N. Thanki, P. D. Thomas, S. C. E. Tosatto, C. H. Wu, A. Bateman, InterPro in 2022. Nucleic Acids Res. 51, D418–D427 (2023). 36350672 10.1093/nar/gkac993 PMC9825450

