# Supplementary material for: Chromatin analysis of adult pluripotent stem cells reveals a unique stemness maintenance strategy
Source: Sci Adv. 2023 Oct 6;9(40):eadh4887. doi: 10.1126/sciadv.adh4887 (PMC10558129; doi:10.1126/sciadv.adh4887)
Supplement: Supplementary file 1 — Figs. S1 to S15 Legends for tables S1 to S3, S6 and S7 Tables S4 and S5 [file sciadv.adh4887_sm.pdf]

Science Advances
AAAS

Supplementary Materials for

Chromatin analysis of adult pluripotent stem cells reveals a unique stemness maintenance strategy

Axel Poulet *et al.*

Corresponding author: Josien C. van Wolfswinkel, josien.van.wolfswinkel@yale.edu

Sci. Adv. **9**, eadh4887 (2023)
DOI: 10.1126/sciadv.adh4887

The PDF file includes:

Figs. S1 to S15
Legends for tables S1 to S3, S6 and S7
Tables S4 and S5

Other Supplementary Material for this manuscript includes the following:

Tables S1 to S3, S6 and S7

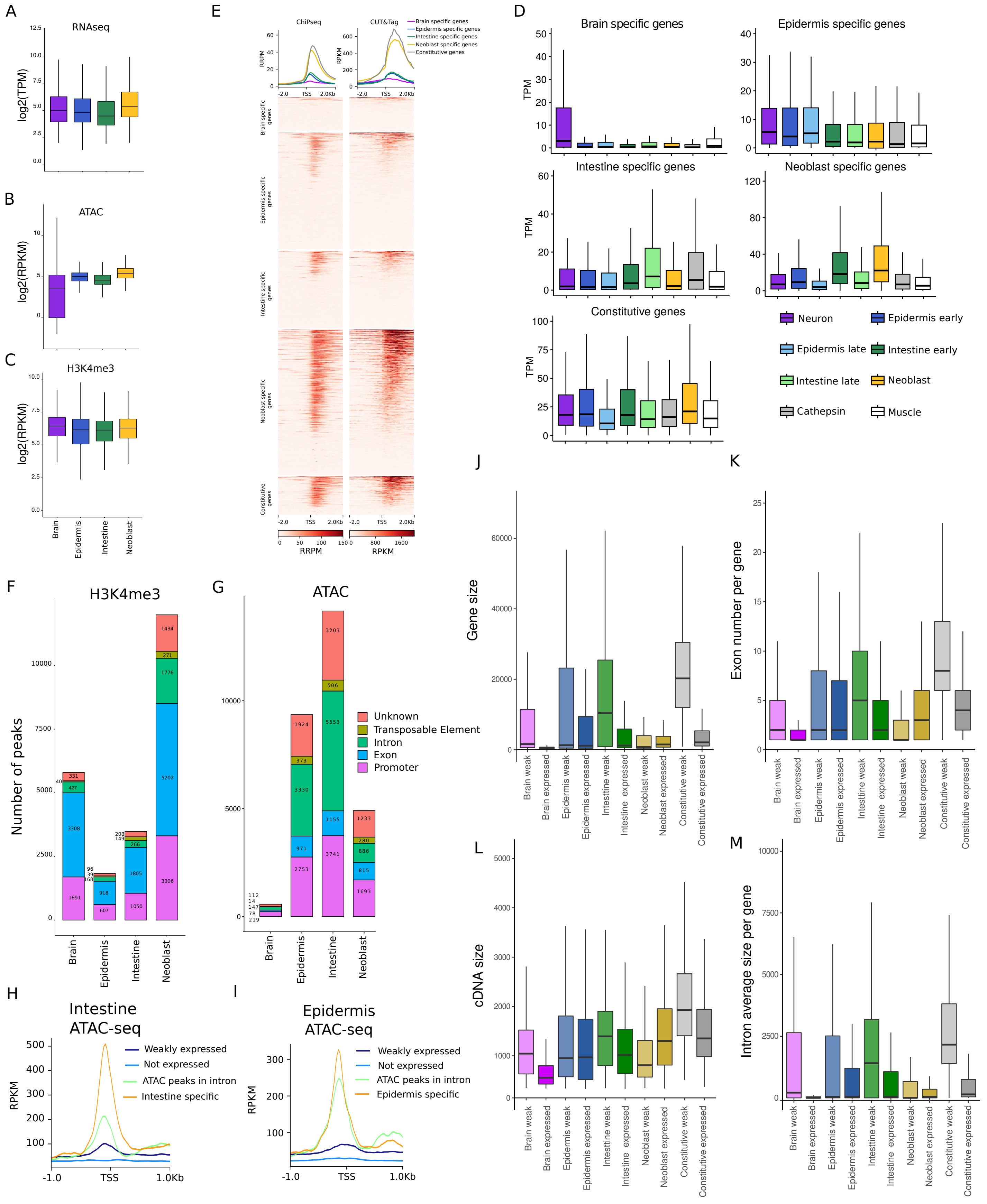

A
RNAseq
log2(TPM)
B
ATAC
log2(RPKM)
C
H3K4me3
log2(RPKM)
Brain
Epidermis
Intestine
Neoblast
E
ChIPseq
CUT&Tag
Brain specific genes
Epidermis specific genes
Intestine specific genes
Neoblast specific genes
Constitutive genes
RRPM
RPKM
TSS
D
Brain specific genes
Epidermis specific genes
Intestine specific genes
Neoblast specific genes
Constitutive genes
TPM
Neuron
Epidermis early
Epidermis late
Intestine early
Intestine late
Neoblast
Cathepsin
Muscle
F
H3K4me3
Number of peaks
G
ATAC
Unknown
Transposable Element
Intron
Exon
Promoter
H
Intestine ATAC-seq
RPKM
Weakly expressed
Not expressed
ATAC peaks in intron
Intestine specific
-1.0
TSS
1.0Kb
I
Epidermis ATAC-seq
RPKM
Weakly expressed
Not expressed
ATAC peaks in intron
Epidermis specific
J
Gene size
K
Exon number per gene
L
cDNA size
M
Intron average size per gene
Brain weak
Brain expressed
Epidermis weak
Epidermis expressed
Intestine weak
Intestine expressed
Neoblast weak
Neoblast expressed
Constitutive weak
Constitutive expressed

Figure S1: Supporting data on the classification of tissue-specific and constitutive gene sets. A. Boxplots showing the expression (log2(TPM)) of the constitutive genes in the RNA-seq data from the different tissues (brain, epidermis, intestine, neoblast). B. Boxplots of the log2(ATAC_RPKM) over the TSS region (± 1kb) of the constitutive genes in the different cell isolations (brain, epidermis, intestine, neoblast). C. Boxplots of the log2(H3K4me3_RPKM) over the TSS region (± 1kb) of the constitutive genes in the different cell isolations (brain, epidermis, intestine, neoblast). D. Expression levels of the various gene clusters in cell types defined by single cell RNA sequencing confirm the assignments (public data processed: PRJNA276084 *(16)*). Note that mature epidermal cells that are enriched in the epidermal isolation are missing from the single cell datasets due to technical incompatibility. E. Average profile plot and heatmaps of H3K4me3 signal over the various gene clusters from ChIPseq (reference-adjusted reads per million: RRPM) and CUT&Tag (RPKM) show comparable distribution of reads. ChIPseq and CUT&Tag shown are from isolated neoblasts (ChIPseq public data reprocessed GSE74169 *(18)*). FG. Location of the H3K4me3 CUT&Tag peaks (F) and ATACseq peaks (G) detected in each cell isolation. The promoter is defined as the 2kb upstream of the TSS. H. Average profile plot of intestine ATACseq (in RPKM) over weakly expressed genes (1 to 10 TPM), non-expressed genes (< 1 TPM), genes with ATACseq peaks in introns, and intestine-specific genes. I. Average profile plot of epidermis ATACseq (in RPKM) over weakly expressed gene (1 to 10 TPM), non-expressed genes (< 1 TPM), genes with ATACseq peaks in intron and epidermis-specific genes. JKLM. Gene size (J), exon number (K), cDNA size (L), and intron size (average per transcript) (M) per gene cluster. Highly expressed genes tend to be smaller than low-expressed genes, except among neoblast-specific genes. The difference largely correlates with larger intron size and higher intron number in the low-expressed genes.

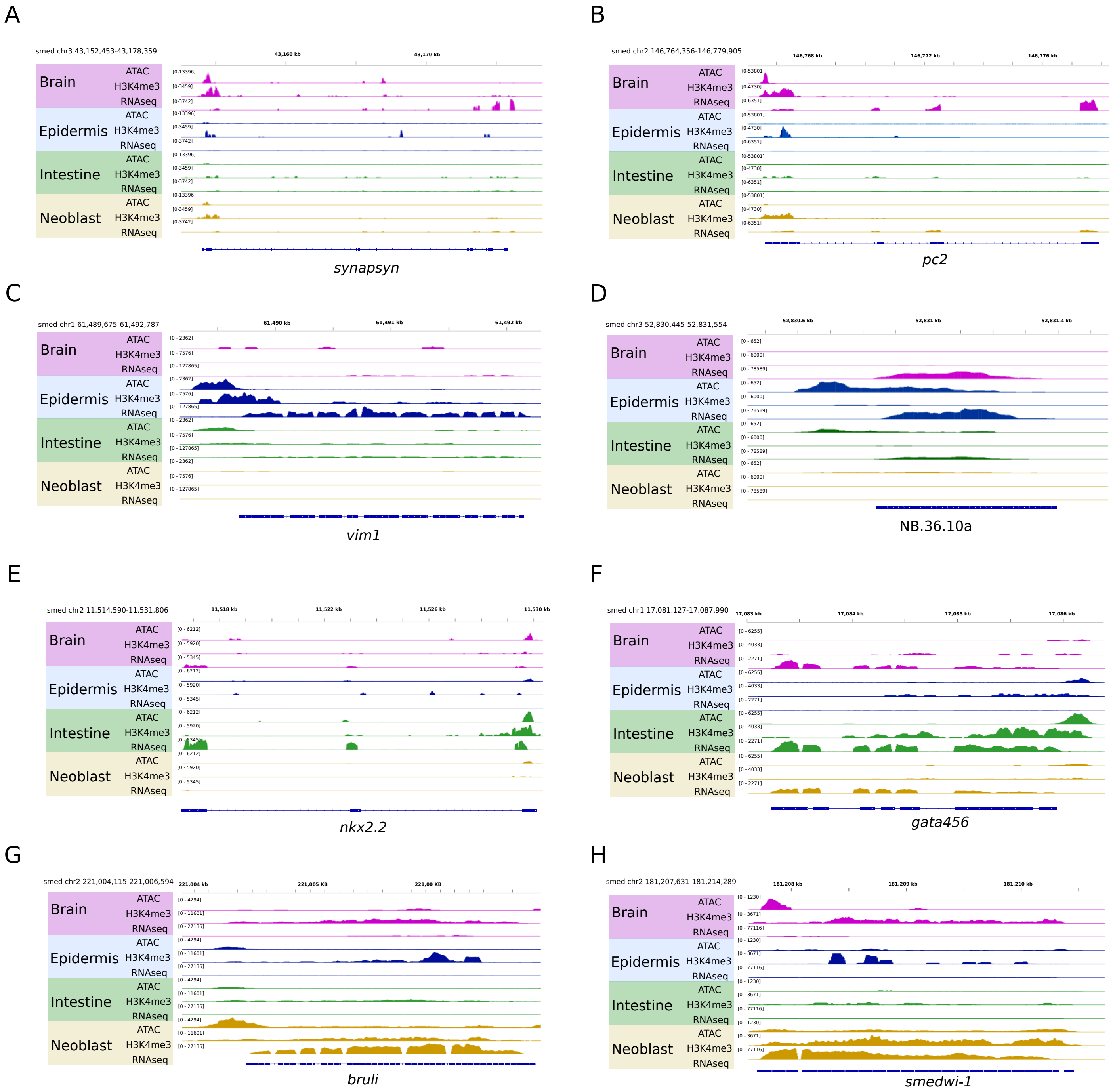

Figure S2: Genome browser views of tissue-specific chromatin and RNAseq data on various known tissue-specific genes. Shown are the RPKM of ATACseq and H3K4me3 CUT&Tag sequencing and the TPM of the RNAseq across tissue-specific genes, for brain, epidermis, intestine and neoblast samples. A. and B. *synapsyn* and *pc2* (neuronal genes). C. and D. *vim1* and *NB.36.10a* (epidermal genes). E. and F. *nkx2.2* and *gata456* (intestinal genes). G. and H. *bruli* and *smedwi-1* (neoblast genes).

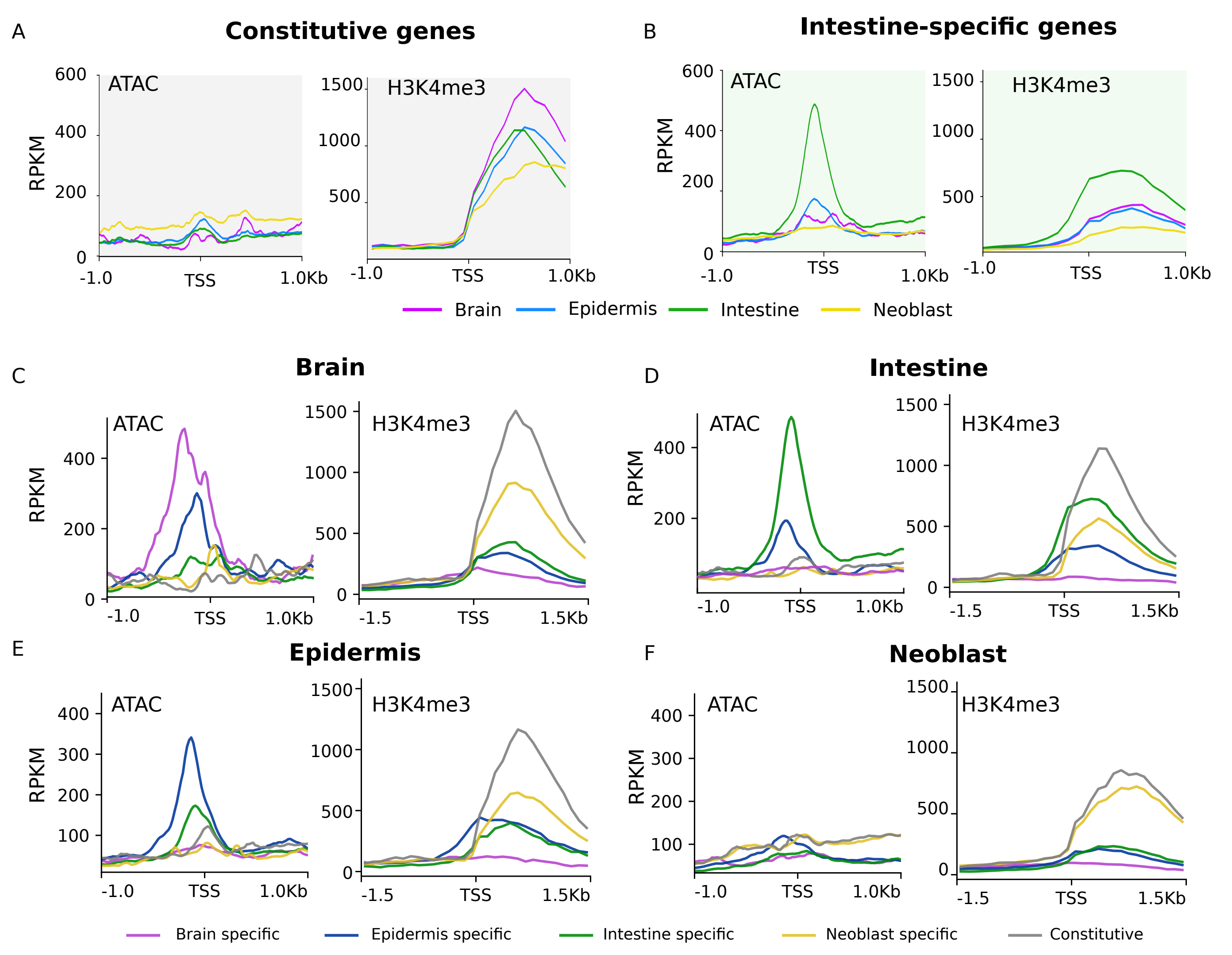

Figure S3: Profile plots of the TSS regions of the different gene clusters. AB. Shown are average RPKM profile plots over the TSS region (± 1kb) of ATACseq (left) and H3K4me3 (right) data from each of the cell isolations for the constitutive gene cluster (A, plot with gray background), and the intestine-specific gene cluster (B, plot with green background). Constitutive genes show a high H3K4me3 signal in the absence of a clear ATACseq peak in each of the isolations. The intestinal genes show an intestine-specific ATAC peak and increased H3K4me3 signal in the intestinal sample. CDEF. Shown are average RPKM profile plots over the TSS region (± 1kb) of ATACseq and H3K4me3 CUT&Tag data for each of the different gene clusters in brain (C), epidermis (D), intestine (E) and neoblast (F) isolations. Each of the differentiated tissues shows an ATAC peak of its tissue-specific genes.

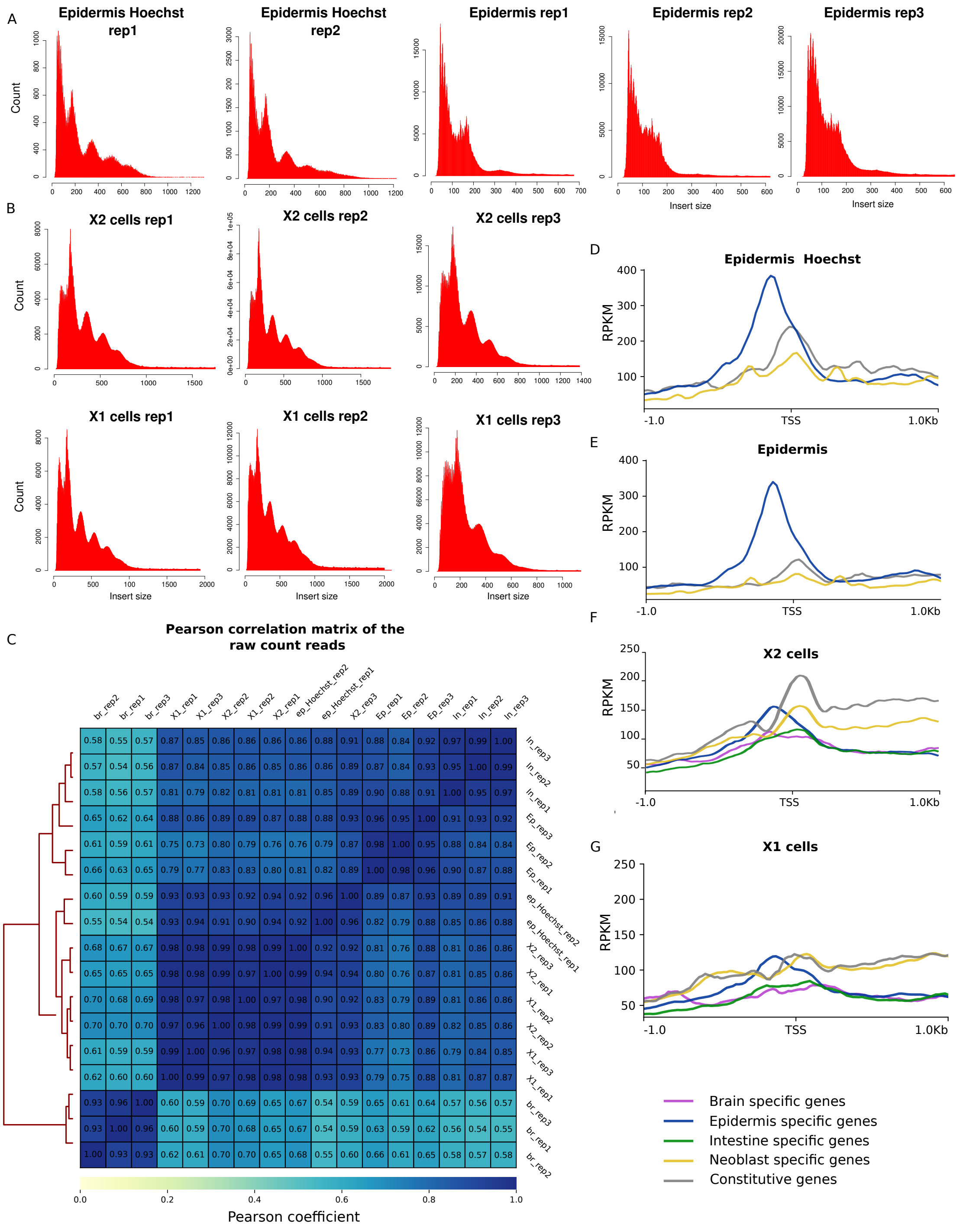

Figure S4: Control experiments to exclude technical artifacts from ATACseq experiments. A. Comparison of the library insert size of epidermis treated with Hoechst versus the epidermis without Hoechst staining (Li et al (PRJNA633618) *(30)*. Hoechst staining does not have a negative effect on library quality. B. Comparison of the library insert size of G1/G0 stem cells (X2) versus G2/M phase neoblast cells (X1). Cells in G2/M phase do not show a siginficantly altered profile compared to cells in G1 phase. C. Pearson correlation matrix of the ATACseq used in this analysis: epidermis, epidermis treated with Hoechst, intestine, brain, neoblast and G1/G0 cells, computed with Deeptools *(82)*. DEFG. Average profile plots (in RPKM) over the TSS region (± 1kb) of epidermis with Hoechst (D), epidermis without Hoechst (E), X2 cells (F), and X1 cells (G). Again, no major effects of Hoechst staining or cell cycle state are detected.

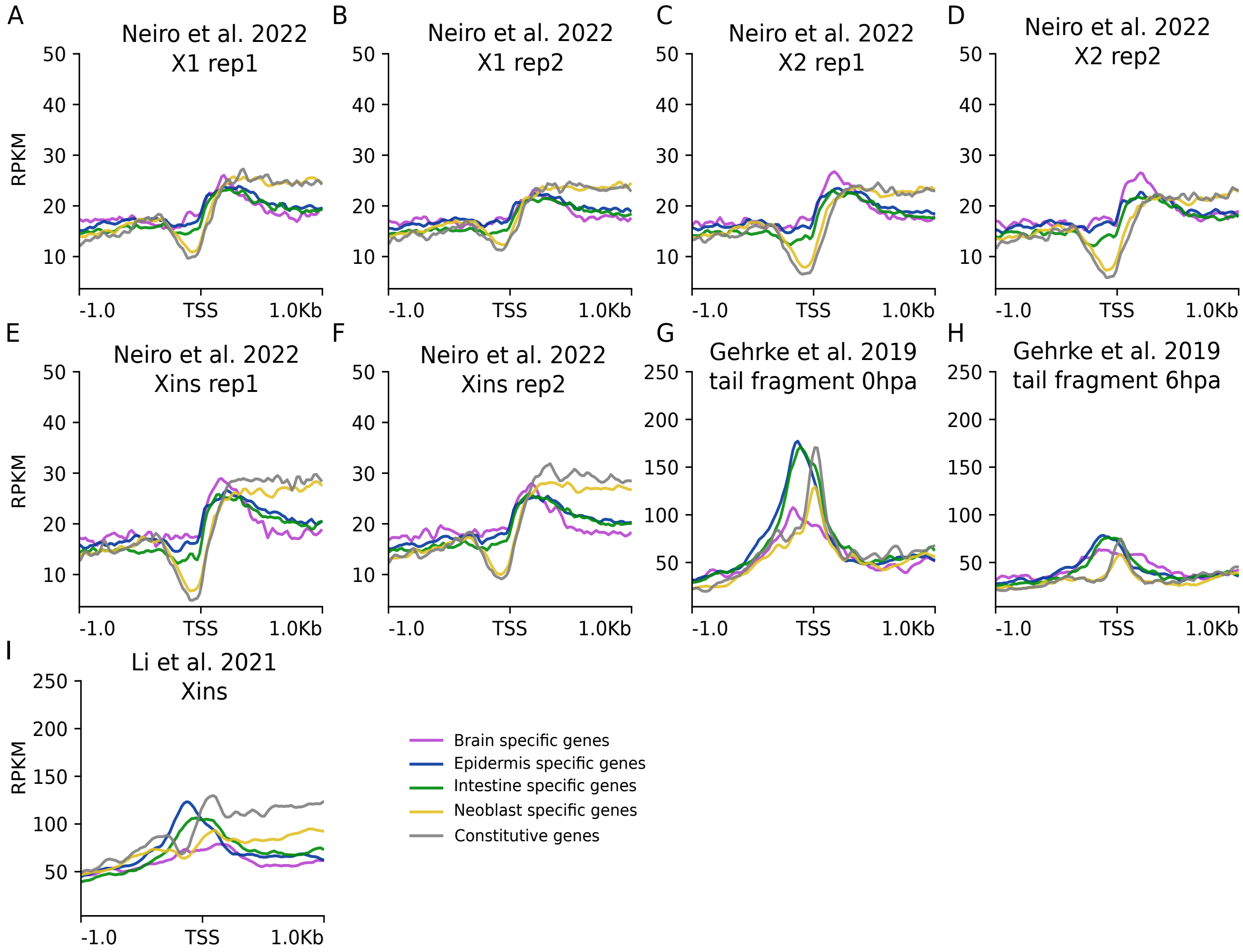

Figure S5: Analysis of publicly available ATACseq datasets. Shown are average RPKM average profile plots over the TSS region (± 1kb) of the gene clusters, using various public datasets. While these datasets are not tissue-specific, the same data processing was applied for all of them. ABCDEF. ATACseq data from Neiro et al. *(31)* derived from neoblasts (A and B), differentiated cells (E and F), and mixed populations of neoblast and differentiated cells (C and D) (PRJNA832235). GH. ATACseq data from Gehrke et al. *(32)* derived from tail fragments of *S. mediterranea* at 0h (G) and 6h (H) after amputation (PRJNA515075). I. ATACseq data from Li et al. *(30)* derived from differentiated cells (PRJNA633618). Throughout all datasets the similarity of neoblast genes and constitutive genes is detected, whereas tissue-specific genes follow a different profile. In data from Gehrke and Li, the shift of the ATAC peak in neoblast genes and constitutive genes is confirmed: only differentiated tissue genes show a peak upstream of the TSS, whereas for neoblast genes and constitutive genes the peak is on top of the TSS.

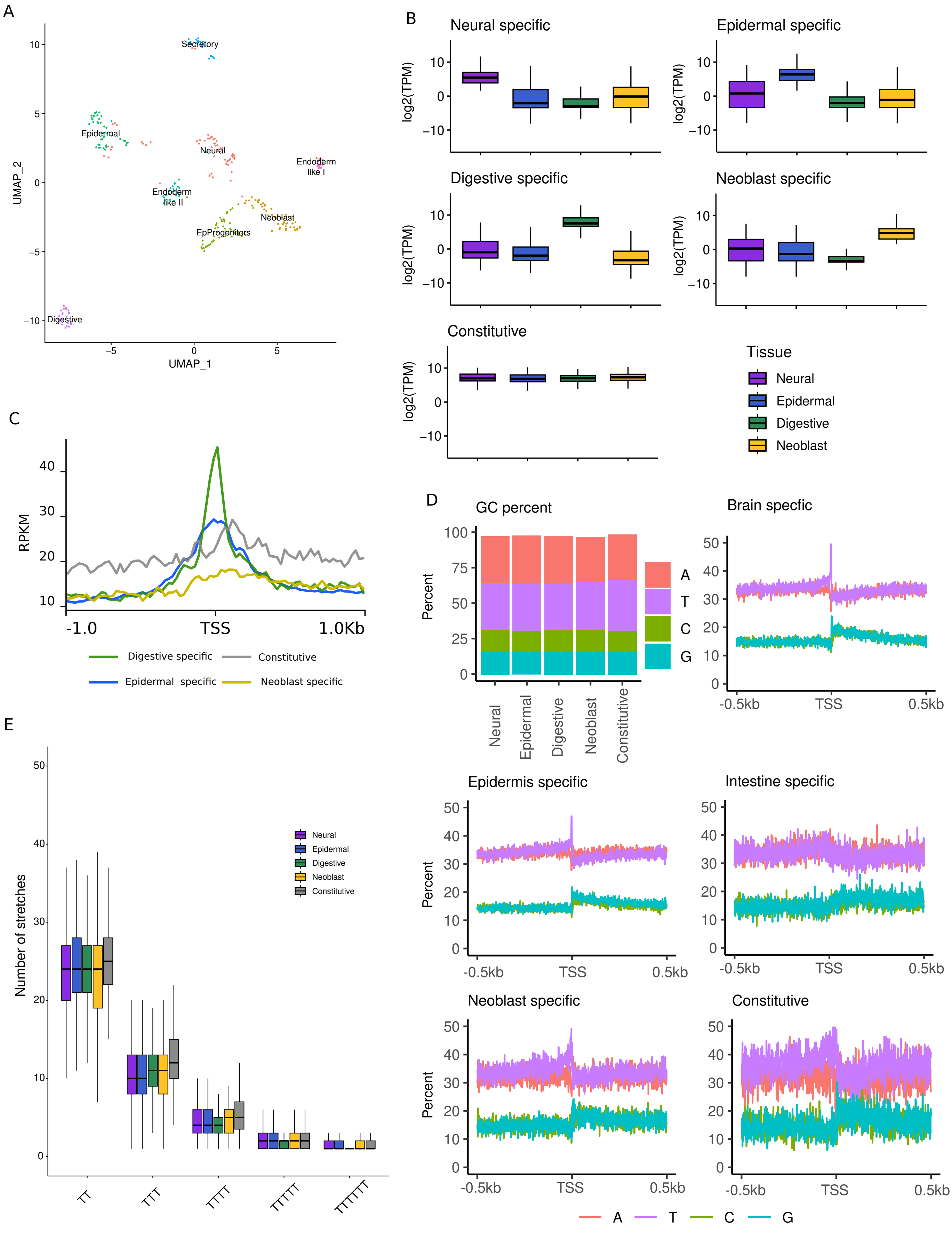

A
Secretory
Epidermal
Neural
Endoderm like I
Endoderm like II
Neoblast
EpProgenitors
Digestive
UMAP_1
UMAP_2
B
Neural specific
Epidermal specific
Digestive specific
Neoblast specific
Constitutive
log2(TPM)
Tissue
Neural
Epidermal
Digestive
Neoblast
C
RPKM
-1.0
TSS
1.0Kb
Digestive specific
Constitutive
Epidermal specific
Neoblast specific
D
GC percent
Percent
A
T
C
G
Neural
Epidermal
Digestive
Neoblast
Constitutive
Brain specfic
Epidermis specific
Intestine specific
Neoblast specific
Constitutive
-0.5kb
TSS
0.5kb
E
Number of stretches
Neural
Epidermal
Digestive
Neoblast
Constitutive
TT
TTT
TTTT
TTTTT
TTTTTT

Figure S6: Analysis of the promoter organization of tissue-specific and constitutive genes in the acoel *Hofstenia miamia*. A. Re-analysis and UMAP of sequenced cells (PRJNA889328) *(33)* of hatchling worms recovers cluster identity similar to results obtained by the original authors, corresponding to identifiable cell types, including neoblasts. These clusters were used for the identification of tissue-specific gene sets. A gene was defined as tissue-specific if it was more than 2-fold enriched in one tissue compared to each of the other tissues. A gene was defined as constitutive if the enrichment for all the tissue comparisons was between 0.5 and 2-fold. Based on these criteria, we identified 373 intestine-specific genes, 1913 epidermis-specific genes, 1631 neuron-specific genes, 408 stem cell-specific genes, and 150 constitutive genes. B. Aggregated expression over the cell types of the various defined clusters of tissue-specific and constitutive genes defined in A. C. Median RPKM profile plots over the predicted TSS region (± 1kb) of tissue-specific and constitutive genes, based on ATACseq datasets of merged tail and head 0h after amputation were reanalyzed (PRJNA512373 *(32)*. Whereas epidermal and intestinal genes have a clear peak in chromatin accessibility around the TSS, neoblast genes and constitutive genes lack this organization. D. Analysis of the AT content over the region surrounding the TSS (-/+ 500 bases around the TSS) of *Hofstenia* gene clusters. The bar plot represents the aggregated percentage of nucleotide content in each gene cluster. The other plots represent the nucleotide representation at each position over the region of interest. Similarly to the situation in *S. mediterranea*, neoblast-specific genes and constitutive genes have a higher T-content in the region upstream of the TSS. E. Quantification of T-stretches observed over the TSS in the different gene clusters. The sequences analyzed correspond to the 500 bases upstream of the TSS and are reverse complemented when genes are on reverse strand. Neoblast genes and constitutive genes have more stretches of 4 or more Ts in their promoter regions. Please note that due to limited data and absence of tissue-specific data the predictions of the TSS positions are suboptimal, and this increased the noise in this analysis.

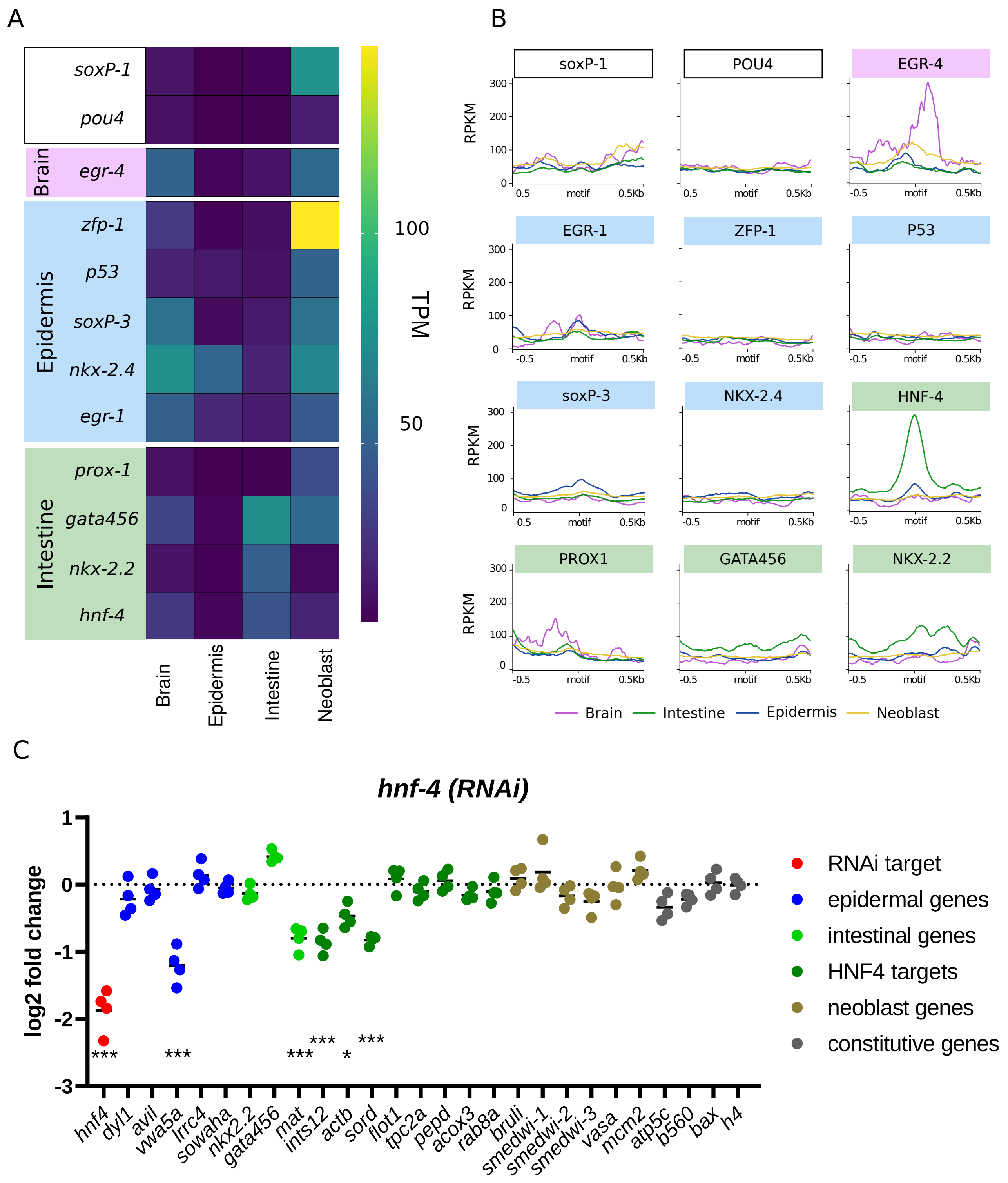

Figure S7: Knockdown of HNF4 affects intestinal gene expression. A. Expression levels of planarian transcription factors in each of the tissue isolations. Many of the transcription factors have high expression in the neoblasts. Intestinal transcription factors are enriched in the intestine. B. Metaplots (average RPKM) depicting chromatin accessibility over several predicted TF binding motifs of interest from panel A (± 500b) in each of the cell isolations. C. RNAi-mediated knockdown of the predicted intestinal transcription factor HNF4 indeed results in reduced expression of a subset of the known intestinal genes (shown in light green) and predicted intestinal HNF4 targets (dark green), with minimal effects on neoblast (yellow), constitutive (grey), and epidermal (blue) genes. Statistical significance is determined using a Student's t-test (p-value: * ≤0.05, ** ≤0.01, *** ≤0.005).

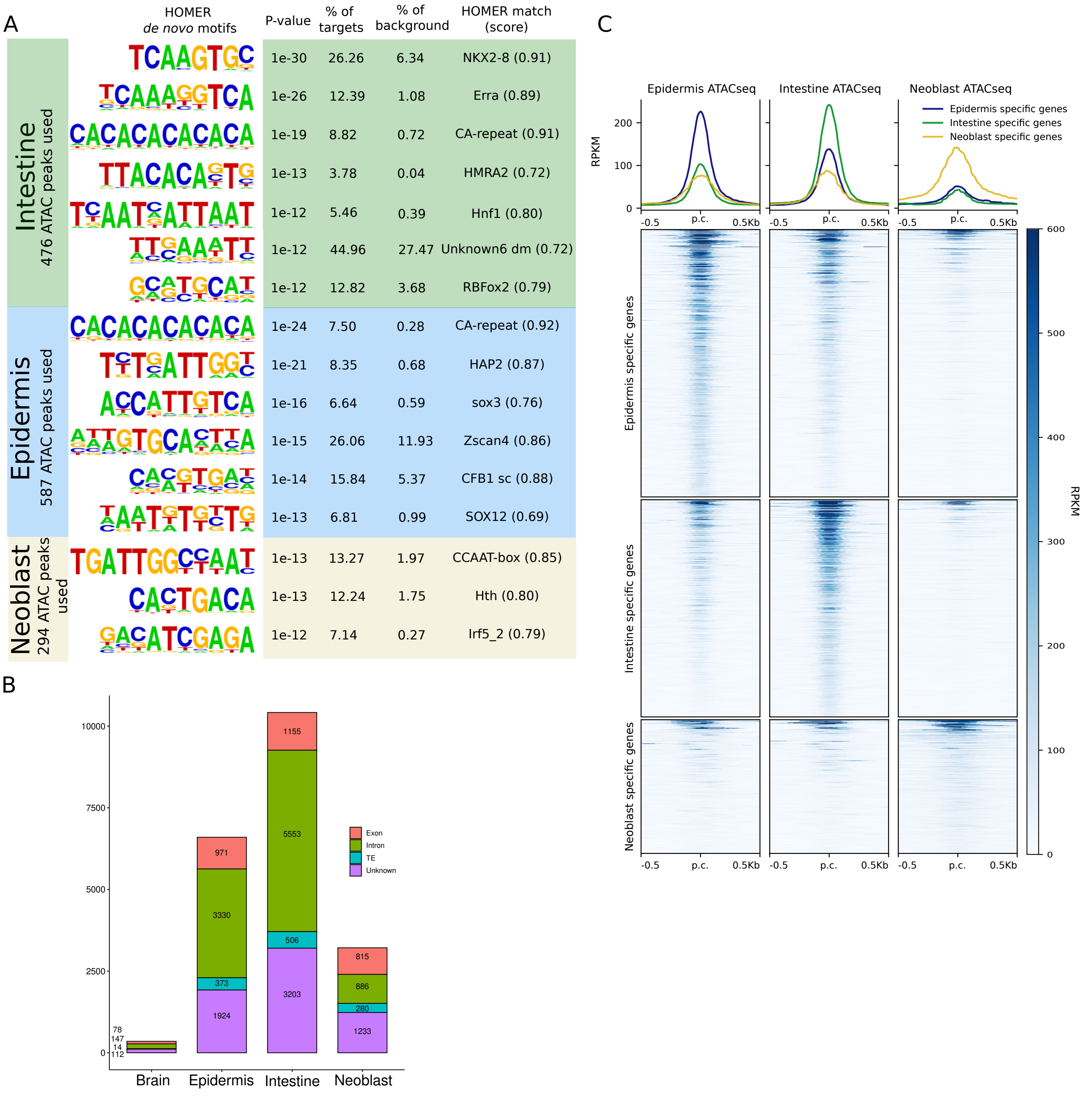

Figure S8: Enhancer motif analysis. A. Enriched motifs in ATACseq peaks not localized in the promoter regions and assigned as putative enhancers of intestinal, epidermal or neoblast specific genes. Putative enhancer regions were assigned to the closest TSS. No significantly enriched motifs were found in putative enhancers in proximity to brain-specific or constitutive genes. B. Non-promoter ATAC peaks are primarily located in introns and intergenic ("unknown") regions. C. Average profile plots and heat maps of accessible regions outside of gene promoters. Shown is accessibility at the non-promoter peaks of either epidermis-specific genes (top group), intestine-specific genes (middle group), or neoblast-specific genes (bottom group), relative to the peak center. Epidermal and intestinal peaks are largely tissue-specific. However, the genes that have enhancer accessibility in neoblasts have relatively broad and weak peaks, and this accessibility tends to be present in other tissue isolations as well.

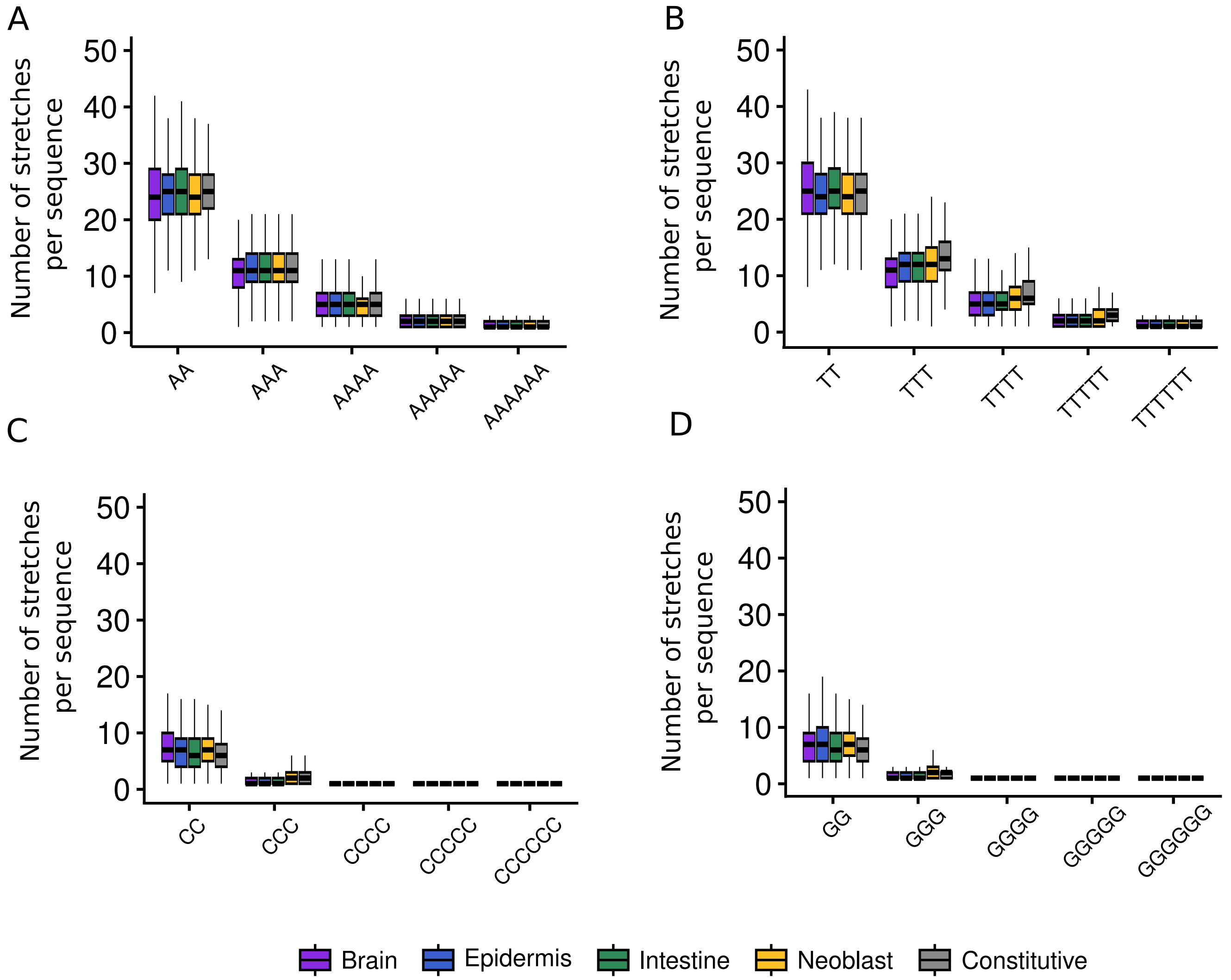

Figure S9: Nucleotide stretches observed over the TSS in the different gene clusters. A. Frequency of stretches of A nucleotides. B. T nucleotides. C. C nucleotides. D. G nucleotides. The sequences analyzed correspond to the 500 bases upstream of the TSS and are reverse complemented when genes are on the reverse strand. A- and T-stretches are more abundant than C- or G- stretches. Only T-stretches are significantly enriched in neoblast and constitutive gene promoter regions.

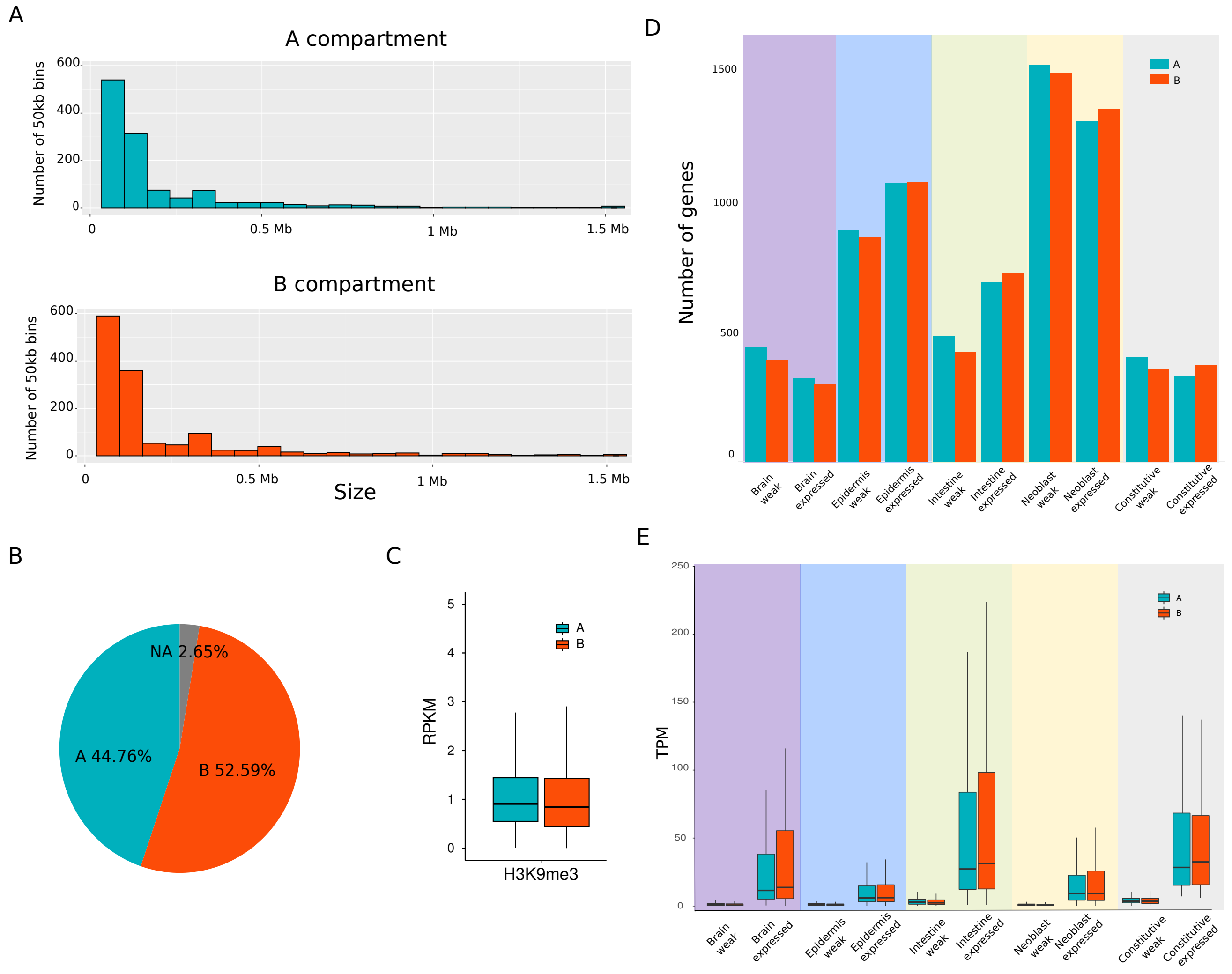

Figure S10: Hi-C interaction maps of A/B compartments. A. Size distribution of the A and B compartment. B. Pie chart of the percentage of 50kb bins annotated as A, B and NA (not attributed) compartments. C. Comparison of the H3K9me3 content between the A and B compartment. D. Distribution of genes between the A and B compartment (weak genes: less than 10 TPM). E. Distribution of gene expression levels in the A and B compartments (weak genes: less than 10 TPM).

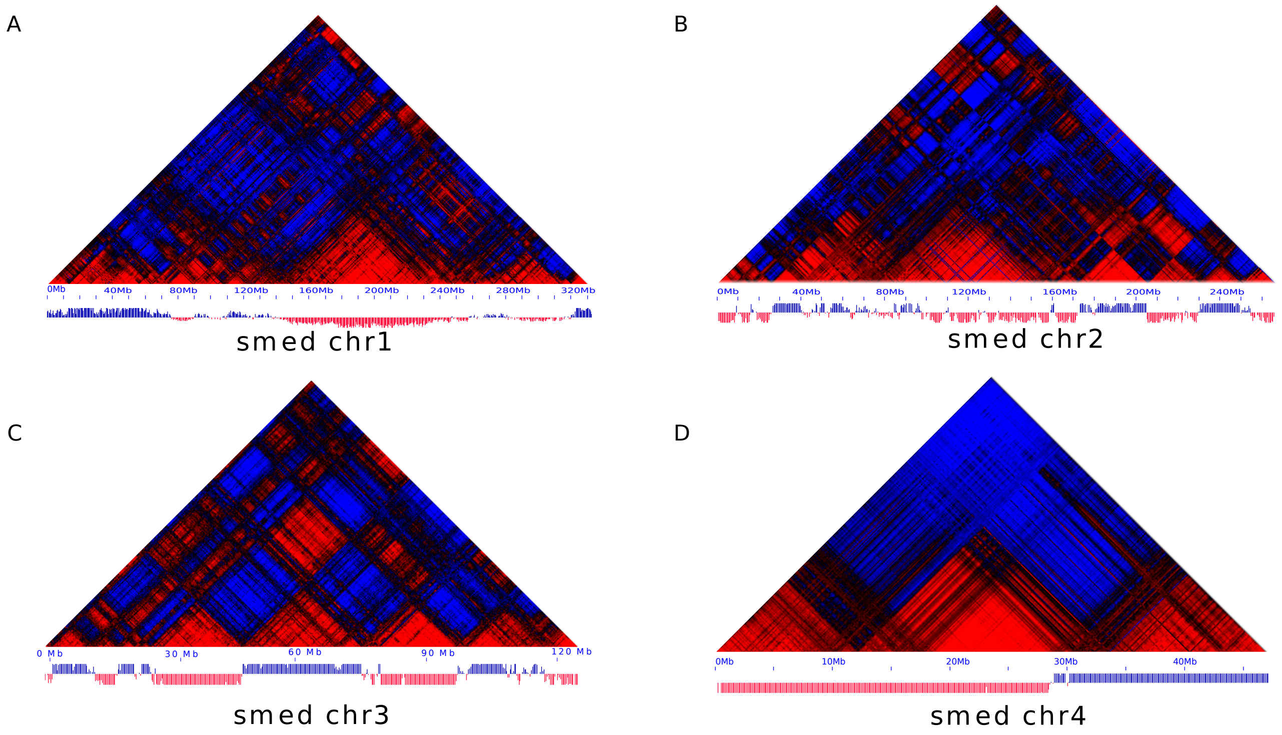

Figure S11: Hi-C interaction maps of A/B compartments per chromosome. ABCD. Pearson correlations and A/B compartment classification of distance-normalized Hi-C interaction frequency maps of the chromosome 1 (A), chromosome 2 (B), chromosome 3 (C) and chromosome 4 (D) of *S. mediterranea* displayed using Juicer *(90)*. The compartments were called at 50kb resolution.

Figure S12: Analysis of A/B compartment features per chromosome. A. Distribution of the genes in A and B compartments per chromosome (related to Supplemental figure 10D). We used the TSS +1 base as the coordinate for each gene. B. Gene expression in TPM over A/B compartment (related to Supplemental figure 10E). C. Chromatin characteristics of A/B compartment (related to Figure 4B and Supplemental figure 10C). The data of ATAC, H3K4me3 and H3K9me3 of the various tissues were merged for this analysis, and the RPKM was computed over the 50kb bins of the A/B compartment annotation. D. Percentage of transposable element annotation per family over A/B compartment compared to the whole genome percentage (in gray) (related to Figure 4D).

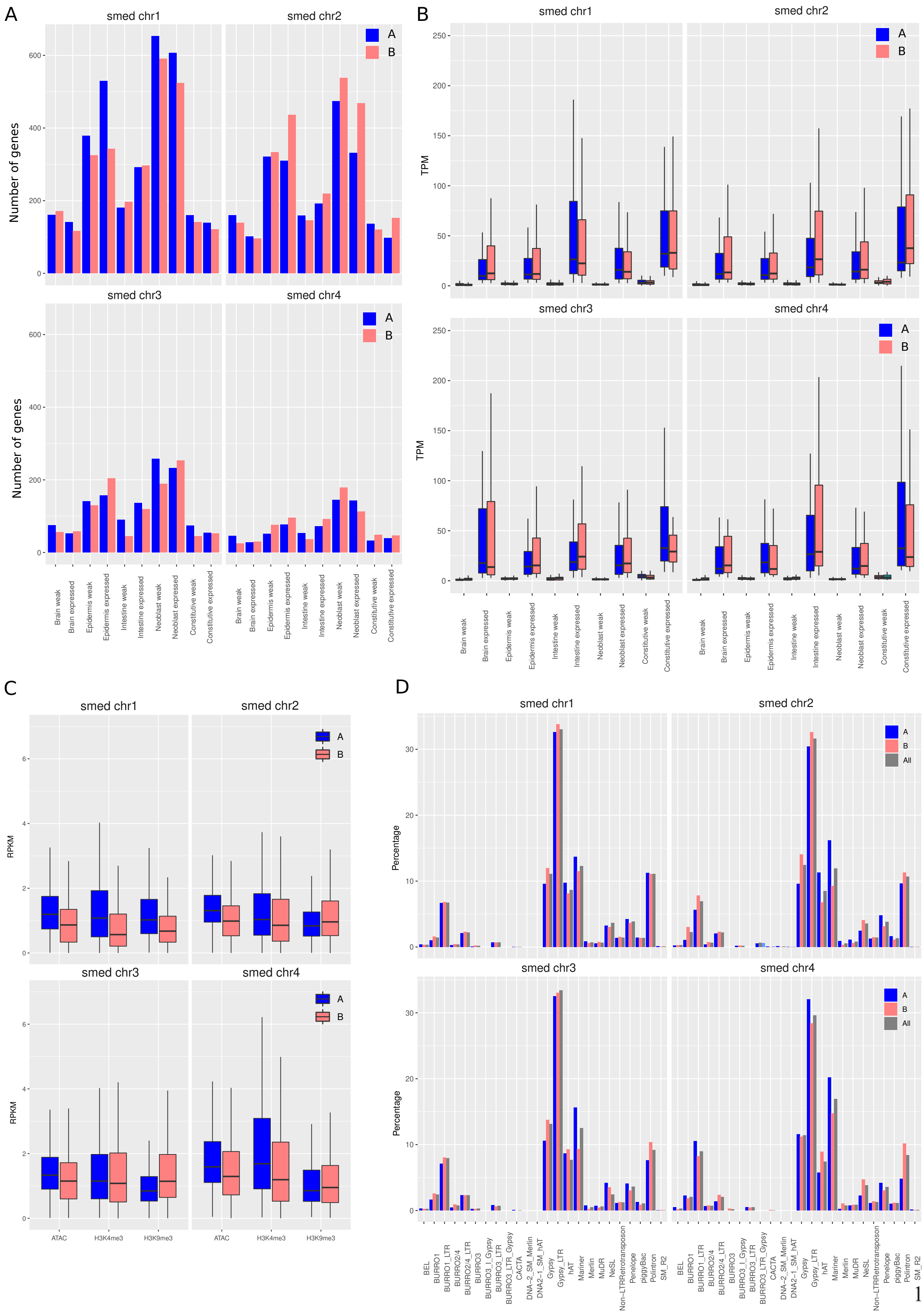
A
smed chr1
smed chr2
smed chr3
smed chr4
Number of genes
A
B
0
200
400
600
Brain weak
Brain expressed
Epidermis weak
Epidermis expressed
Intestine weak
Intestine expressed
Neoblast weak
Neoblast expressed
Constitutive weak
Constitutive expressed
B
TPM
0
50
100
150
200
250
C
RPKM
0
2
4
6
ATAC
H3K4me3
H3K9me3
D
Percentage
0
10
20
30
All
BEL
BURRO1
BURRO1_LTR
BURRO2/4
BURRO2/4_LTR
BURRO3
BURRO3_I_Gypsy
BURRO3_LTR
BURRO3_LTR_Gypsy
CACTA
DNA−2_SM_Merlin
DNA2−1_SM_hAT
Gypsy
Gypsy_LTR
hAT
Mariner
Merlin
MuDR
NeSL
Non−LTRRetrotransposon
Penelope
piggyBac
Polintron
SM_R2

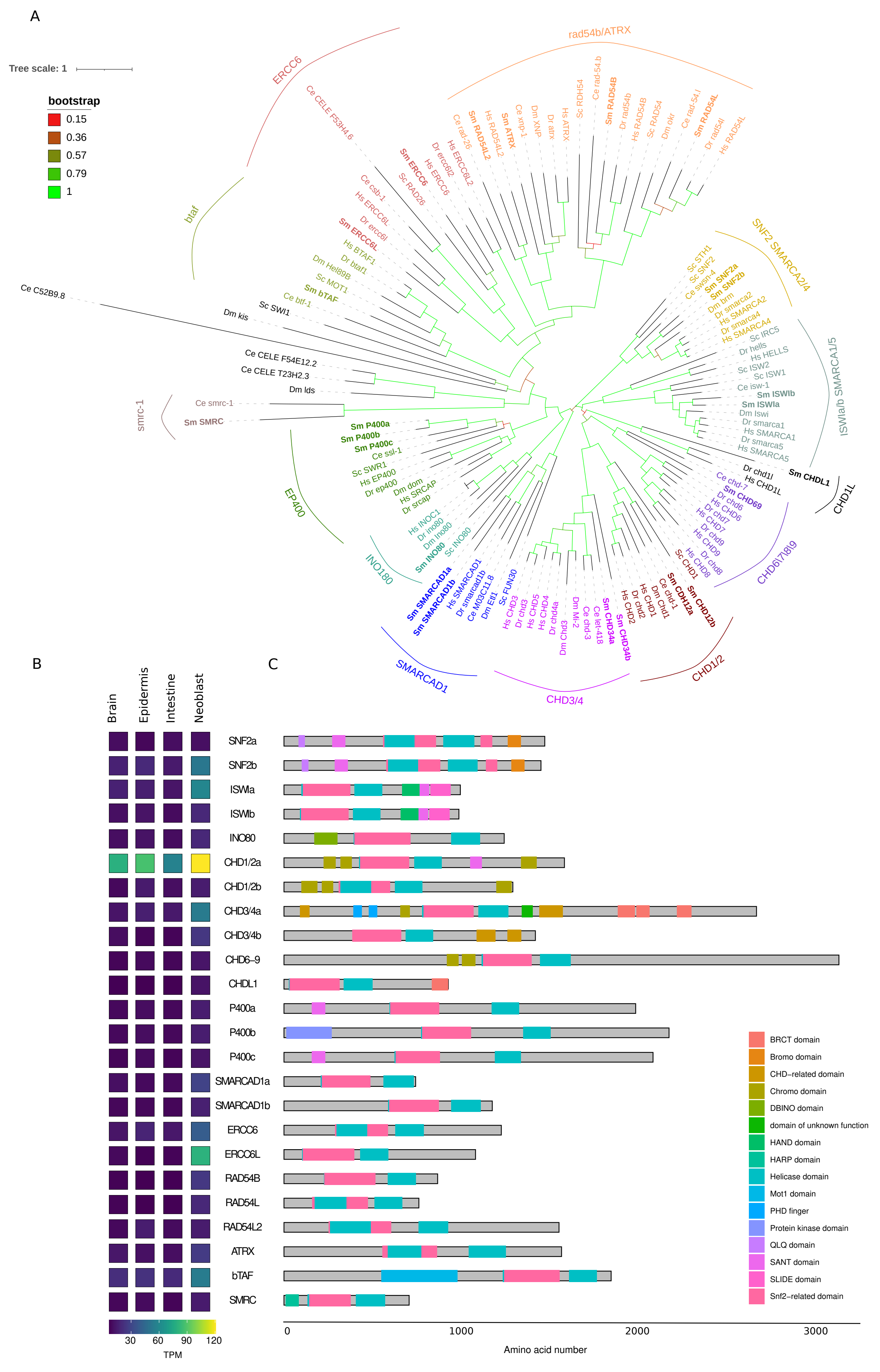

A
Tree scale: 1
bootstrap
0.15
0.36
0.57
0.79
1
B
Brain
Epidermis
Intestine
Neoblast
TPM
C
Amino acid number
BRCT domain
Bromo domain
CHD–related domain
Chromo domain
DBINO domain
domain of unknown function
HAND domain
HARP domain
Helicase domain
Mot1 domain
PHD finger
Protein kinase domain
QLQ domain
SANT domain
SLIDE domain
Snf2–related domain

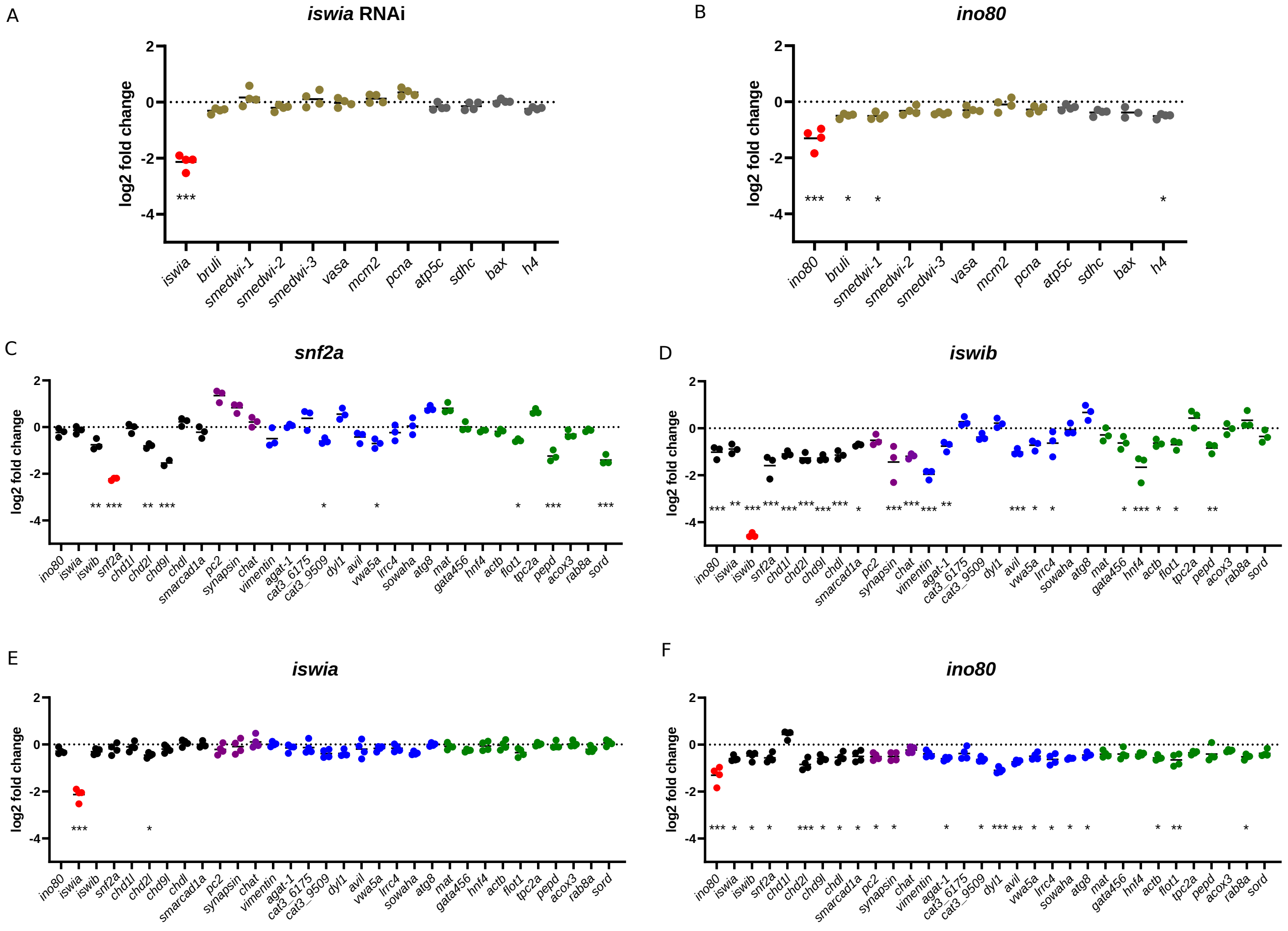

Figure S14: Effect of remodeler knockdown on gene expression. AB. qPCR data for neoblast-specific (yellow) and constitutive (grey) genes upon reduction of ISWIa (A) and INO80 (B) shows no major effects on gene expression. CDEF. qPCR data for chromatin remodelers (black), neuronal (purple), epidermal (blue), and intestinal (green) genes upon reduction of SNF2 (C), ISWIb (D), ISWIa (E), and INO80 (F). Statistical significance is determined using a Student's t-test (p-value: * ≤0.05, ** ≤0.01, *** ≤0.005).

Figure S13: Overview of planarian chromatin remodelers. A. Phylogenetic tree of the proteins with SNF2-like domains (*S. mediterranea* proteins are indicated in bold). The color of the branches indicates the bootstrap score. Ce: *Caenorhabditis elegans*; Dr: *Danio rerio*; Dm: *Drosophila melanogaster*; Hs: *Homo sapiens*; Mm: *Mus musculus*; Sc: *Saccharomyces cerevisiae*; Sm: *Schmidtea mediterranea*. B. Heatmap of RNAseq-based expression levels of the genes related to SNF2. The color scale depicts the TPM, with blue representing the lowest expression and yellow representing the highest. C. Schematic of protein domains of the *S. mediterranea* chromatin remodeler orthologues made with Interproscan *(97)*.

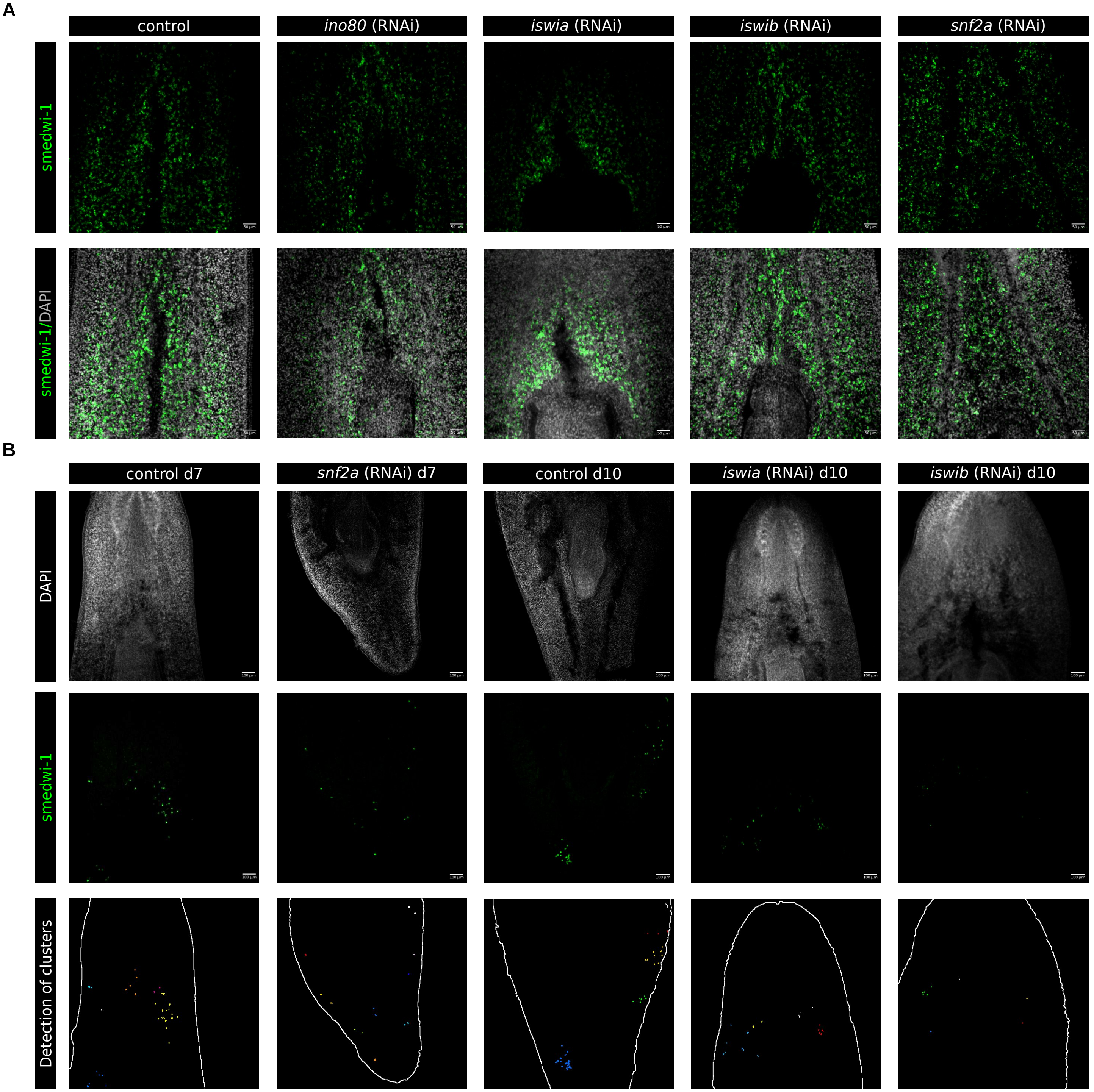

Figure S15: Effect of chromatin remodelers on neoblast expansion. A. Representative images of neoblasts (marked by *smedwi-1* transcript) for the different RNAi conditions under homeostatic conditions. Animals were treated with RNAi for 3 feeds, fixed, and subjected to FISH staining. Shown is a z-projection of 3D confocal images, showing *smedwi-1* (green) and DAPI (gray). Scale bar is 50µm. B. Representative images of neoblasts and the cell clusters as detected by the automated analysis. Animals were treated with RNAi for 3 feeds, and irradiated with 1250Rads to reduce the number of neoblasts. Animals were then grown for 7 or 10 days allowing the remaining neoblasts to expand into colonies. Shown is a z-projection of the 3D confocal images, showing DAPI (top row, gray), *smedwi-1* (middle row, green), or the results of the cluster detection (bottom row, each color represents a different cluster). Scale bar is 100µm.

Table S1. (separate file)
Gene ontology analysis of the best human orthologues of identified tissue-specific genes, analyzed with DAVID.

Table S2. (separate file)
Predicted sequence motifs for markers of interest used to scan with FIMO over TSS of the genes (-2kb to the TSS) of the different gene clusters.

Table S3. **(separate file)**
Probability matrix of the putative Initiator motif as obtained by HOMER.

Table S4.
Sequences of the primers used in this study.

Gene	F Primer	R Primer	Application
snf2	ACAAATTACCGGAGTTGTGGG	CTAGTTTCTCGCGCGCATTC	cloning
ino80	ACGATAGCATTTCTCGCAACA	CGAGTCGATGAGCTCTGTCC	cloning
iswia	ACCGAACAAGAAGAAGAGGAAGA	TGGTTGCTTAGGTGGTCGAG	cloning
iswib	TGCCAGACGAGTTCTCATCT	TGCCAAACGAACTGCCAAT	cloning
hnf4	AGCTTCGATTGTAAACTTCACCA	TTGAGGCCGCTTCAGGTG	cloning
snf2	GGAAAAGAAAAATCAATGAAGGAAGCTCA	CTTAGGCTGCCTGGGGGAC	qPCR - chromatin remodeler
ino80	TCGCTACCAGAATTTCAATGATGATGA	ACCACGCCGCCATTCTAGAA	qPCR - chromatin remodeler
iswia	GGTGGTCGAGGAGCTTTTGG	GGATGGATGGATTCAACCTCCGA	qPCR - chromatin remodeler
iswib	TCGGCTGATGTCAAATTAATACCCA	TGACAGACAGATTTCTATTGATGACTACA	qPCR - chromatin remodeler
chd1l	GCCATTTAGTCGACAATAGTGGCT	CCATTTCACCGGCTGTCATGT	qPCR - chromatin remodeler
chd9l	GCCTACACCGAAAATTCAGAGGTTA	ACTGGTAATCCCGGAGAACGT	qPCR - chromatin remodeler
chdl	TCCATTTCACATAGAACGACAAATCCA	ACTGAAACTCTGAAAACCGGGC	qPCR - chromatin remodeler
smarcad1a	AGGCATCAATTTGACATCGGCA	ACATCTCTTCATTTGTCCCAGTCTATGA	qPCR - chromatin remodeler
bruli	ATGATATTACAGCAATTTCCACATGCCA	GCGGAGGTGTTAAATTTGGGCT	qPCR - neoblast marker
smedwi-1	GTCTCAGAAAACAACTAAAGGTACAGCA	TGCTGCAATACACTCGGAGACA	qPCR - neoblast marker
smedwi-2	ATGGAGGAAATACCAGTAAAAGTAGCT	TTCTAAATGTTCTTCTCAGGCCACC	qPCR - neoblast marker
smedwi-3	GCTACCGGGATTATTTTAAGAACACG	TGATCCTTTGGGACCTCGGT	qPCR - neoblast marker
vasa	CGACTGCCGATGCTGGAGAT	CCTCATGGCATGCGCACAAA	qPCR - neoblast marker
mcm2	TAACATCAATTTCATCTCCAGGCTTGC	GAGAGTCCCGGTACTGTTCCG	qPCR - neoblast marker
pcna	AGTATGCTTCTTCGAAGTGATGGTT	GCGCCTTTAAAATTTTTGAAACACTTGTTA	qPCR - neoblast marker
atp5c	GGTCATGGAGCCAAAGCGTT	ACTATTGGCTGCCCCACACA	qPCR - constitutive gene
sdhc	CCCAGTCCTCCCCATGCTATG	AGGGTCCCAGGCCATATTCG	qPCR - constitutive gene
bax	TGGCTAAACCGGACATATTAATGAAG	CGCCGTTGTTGTGGTATTGG	qPCR - constitutive gene
h4	TGTGACAGCATCGCGGATGA	CGTTTAGCAAGAAGAGGTGGCG	qPCR - constitutive gene
pc2	TGCGCGCTATTGTTGAAGGT	GCTGGCAGCATATCCATCGC	qPCR - brain marker
synapsin	CCCGGAAGTATTTGGACCAATCTG	GCCGTGGATTTTTCGCAGCT	qPCR - brain marker
chat	TTTAATAGCCGGAATCTTGGATTA	GATTATGAGTAGCACGATCCACTG	qPCR - brain marker
vimentin	AACCGCGGCTTCAACTGAAC	CAGCGGAACTTAAAACTCGCTCTT	qPCR - epidermal marker
agat-1	CCTAAAAGGCGAAGGTGTGACT	TGCAACATCCAAACCGACAGA	qPCR - epidermal marker
cat3_6175	TGTTCTCCACGATGTGACTTGGA	TTGCCGCATATCCACCGTTG	qPCR - epidermal marker
cat3_9509	AATGTTGAATCACGACAAACGGCT	AAGCTGACACGCACCAGTTG	qPCR - epidermal marker
dyl	AGTAACAAATGTCGTGAATTGTTTTCCA	TGCGAATCAGCAAAAGACCGA	qPCR - epidermal marker
avil	AGATCCACTGGCATCAGATATTCTAAAA	ACATTTATCAGATTTACAACCCGACGA	qPCR - epidermal marker
vwa5a	GTTCGAAATGCCAAGGAGCTCA	CGTGCTGTGGACAATGCCAT	qPCR - epidermal marker
lrrc4	TGCAGAATTTTCAGACTAGATTGTCAGA	TTATGCAGCTTATTTCAACCAATACCAGA	qPCR - epidermal marker
sowaha	CCACCAGCAAAACCCTTGTGA	ACTCTGAAAGAAGAAAAGGGGCTT	qPCR - epidermal marker
atg8	ACAACGTTATTCCGCCAACAAGTT	ACACTTTCATCACTGTACGCAACA	qPCR - epidermal marker
mat	AGGCGAAGGACATCCAGATAAAAT	ACGTCTCACAAGCCACTTTAGC	qPCR - intestinal marker
gata456	ACAAACAATCAAATTCCAAGCAGCA	TGACATCGCAATTGGAACCGA	qPCR - intestinal marker
hnf4	CCCGAATCTACCCGGCTTGT	TGATCCGAGAGTCAGCGAGTC	qPCR - intestinal marker
actb	AGCCCCTGAAGAACATCCAGT	GCGGGCGCATTGAATGTTTC	qPCR - intestinal marker
flot1	CTTTGAATTTTCTGAAAAGGAGGAAAAACA	TCCTAATGAAATTTTAGTTGTTTCTGGTGT	qPCR - intestinal marker
tpc2a	TGAACAAATGTGGTCTACTCCAACT	GCAACATGACCATTCGTATTCTGC	qPCR - intestinal marker
pepd	CCACGTAAAAGGCCAGCTCC	CGGCCTCCAGTGCAGTTAAA	qPCR - intestinal marker
acox3	TGCTCATAGACTTGTCCATCGGA	TGAATTGAAACCGGACGCTGT	qPCR - intestinal marker
rab8a	GTCGCTGTTGGCATGCTCAG	TCGTGGAGCTACTGGAATAATGCT	qPCR - intestinal marker
sord	GGTCGAATAGGGGATTTTGTTGTCG	AGCCACTCGATCTCCTTCTTTCA	qPCR - intestinal marker
nkx2.2	TCGCCAAATTATGCTCAGATTGATCC	CGATTTCTTGTTCACTGGAATTCGGA	qPCR - intestinal marker
ints12	TGTGCAGTGCTGGTATAGTGGA	TCGGAATTTCAGAGCGTCGGA	qPCR - intestinal marker

Table S5.
Number of genes per cluster and average TPM in the different tissues.

	Number of Genes	Brain avg TPM	Epidermis avg TPM	Intestine avg TPM	Neoblast avg TPM
**Brain expressed**	631	202.79	29.63	26.01	11.48
**Brain weak**	850	3.36	0.50	0.44	0.66
**Epidermis expressed**	2208	51.11	201.08	39.10	9.68
**Epidermis weak**	1823	1.05	5.03	1.10	1.01
**Intestine expressed**	1451	107.99	74.33	373.23	14.46
**Intestine weak**	921	2.01	1.94	4.07	0.79
**Pharynx expressed**	953	43.54	12.12	22.03	13.36
**Pharynx weak**	1082	0.65	0.26	0.32	0.53
**Neoblast expressed**	2733	83.79	16.03	38.10	230.17
**Neoblast weak**	3090	0.60	0.46	0.38	4.23
**Constitutive expressed**	725	105.61	93.94	80.77	127.84
**Constitutive weak**	774	3.49	4.20	3.03	5.10
**other expressed**	2146	97.96	118.73	65.54	67.11
**other weak**	17561	1.41	1.69	1.00	2.27
**NA NA**	37289	0.07	0.11	0.05	0.28

Table S6. (separate file)
Complete listing of the genes that make up the various S. *mediterranea* gene clusters

Table S7. (separate file)
Complete listing of the genes that make up the various *H. miamia* gene clusters
